# 2.7 Å cryo-EM structure of vitrified *M*. *musculus* H-chain apoferritin from a compact 200 keV cryo-microscope

**DOI:** 10.1371/journal.pone.0232540

**Published:** 2020-05-06

**Authors:** Farzad Hamdi, Christian Tüting, Dmitry A. Semchonok, Koen M. Visscher, Fotis L. Kyrilis, Annette Meister, Ioannis Skalidis, Lisa Schmidt, Christoph Parthier, Milton T. Stubbs, Panagiotis L. Kastritis

**Affiliations:** 1 ZIK HALOmem, Charles-Tanford-Proteinzentrum, Martin Luther University Halle-Wittenberg, Halle/Saale, Germany; 2 AIMMS Division of Molecular Toxicology, Faculty of Science, Vrije Universiteit Amsterdam, Amsterdam, The Netherlands; 3 Institute of Biochemistry and Biotechnology, Charles-Tanford-Proteinzentrum, Martin Luther University Halle-Wittenberg, Halle/Saale, Germany; 4 ZIK HALOmem, Biozentrum, Martin Luther University Halle-Wittenberg, Halle/Saale, Germany; Centro Nacional de Biotecnologia (CNB-CSIC), SPAIN

## Abstract

Here we present the structure of mouse H-chain apoferritin at 2.7 Å (FSC = 0.143) solved by single particle cryogenic electron microscopy (cryo-EM) using a 200 kV device, the Thermo Fisher Glacios^®^. This is a compact, two-lens illumination system with a constant power objective lens, without any energy filters or aberration correctors, often thought of as a “screening cryo-microscope”. Coulomb potential maps reveal clear densities for main chain carbonyl oxygens, residue side chains (including alternative conformations) and bound solvent molecules. We used a quasi-crystallographic reciprocal space approach to fit model coordinates to the experimental cryo-EM map. We argue that the advantages offered by (a) the high electronic and mechanical stability of the microscope, (b) the high emission stability and low beam energy spread of the high brightness Field Emission Gun (X-FEG), (c) direct electron detection technology and (d) particle-based Contrast Transfer Function (CTF) refinement have contributed to achieving high resolution. Overall, we show that basic electron optical settings for automated cryo-electron microscopy imaging can be used to determine structures approaching atomic resolution.

## Introduction

**Fig 1:**
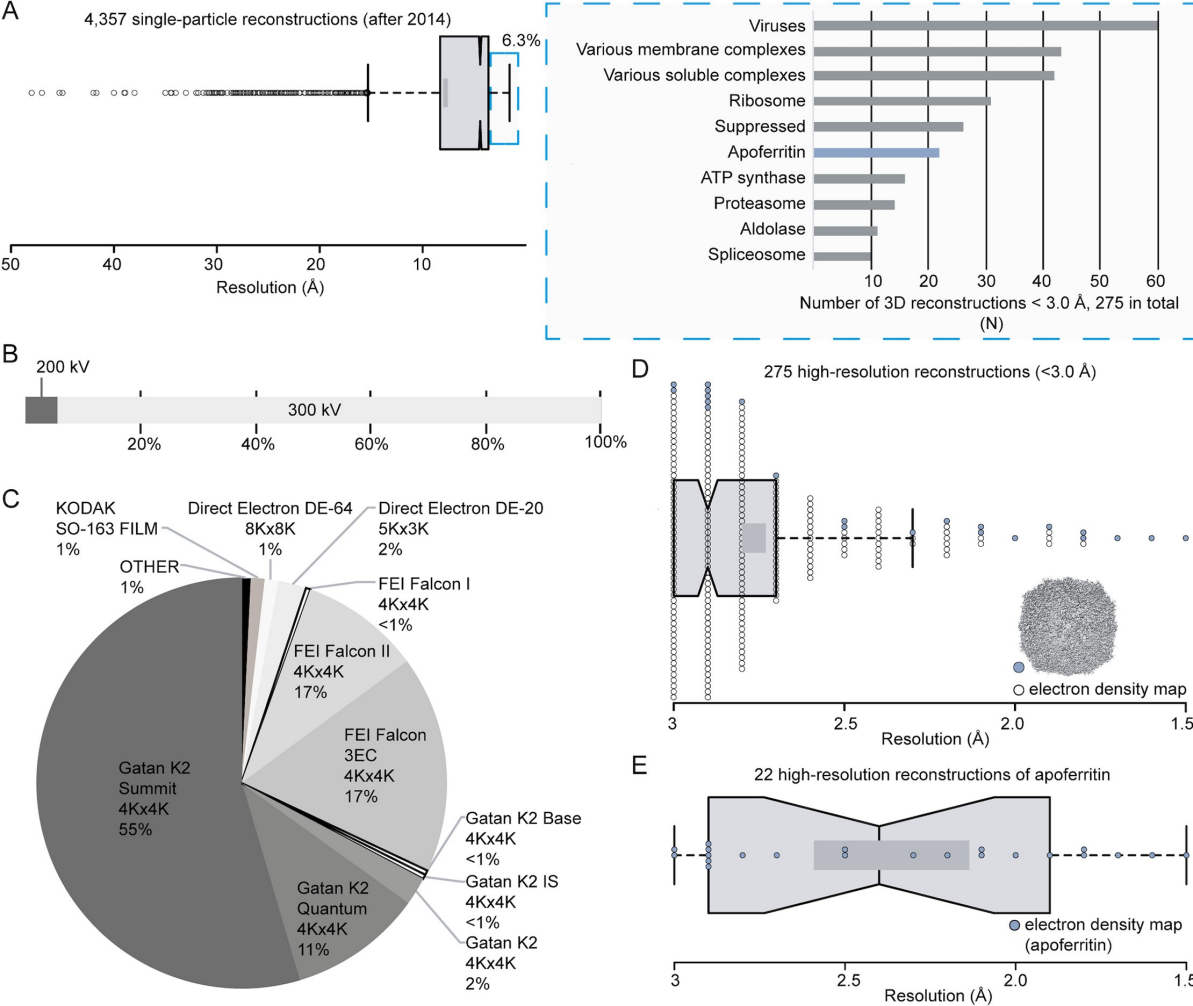
Analysis of single-particle cryo-EM structures from EMDB. (A) On the left, box plot shows the distribution of resolution in all single-particle reconstructions after 2014 and on the right, a bar plot shows the type of specimen reaching resolution better than 3.0 Å; (B) Percentage of structures reaching resolution better than 3.0 Å according to the electron microscope accelerating voltage used to acquire micrographs; (C) A pie chart showing the detector types applied for deriving molecular structures better than 3.0 Å; (D) A box plot showing the distribution of resolution for structures better than 3.0 Å; apoferritin reconstructions are highlighted in blue; (E) A box plot showing the distribution of resolution for apoferritin reconstructions. Dark grey rectangles in (D) and (E) show the confidence intervals at 83%; middle black line shows the average. Data points are shown with circles.

Single-particle cryogenic electron microscopy (cryo-EM) has revolutionized high-resolution structure determination of biomolecular assemblies [1]. The first high-resolution structure better than 3 Å was resolved in 2014 (EMD-6224) and communicated the following year [2]. The vast majority (94%) of protein complexes solved by cryo-EM since 2014 are at a resolution lower than 3 Å (Fig 1A); as of July 2019, only 275 high-resolution (better than 3.0 Å) cryo-EM reconstructions have been deposited in the Electron Microscopy Data Bank (EMDB, https://www.ebi.ac.uk/pdbe/emdb/), for which 194 atomic models (70.5%) are available in the Protein Data Bank (PDB, https://www.rcsb.org/).

Statistical analyses on the 275 reconstructions demonstrate a preference towards specific biomolecules systematically reaching high resolution, with 60% relating to 7 types of molecules (Fig 1A). 164 of these reconstructions are symmetric (S1 Fig), of which half are isometric (59 icosahedral, 25 octahedral and 2 tetrahedral). The overwhelming majority of high-resolution reconstructions (95%) have been resolved using high-end, automated 300 keV electron microscopes (Fig 1B), equipped with direct electron detectors (98%) (Fig 1C), energy filters and constant-power condenser electromagnetic lenses. The high frame rates of direct detection devices (DDD) allow compensation for particle motion (in particular electron beam induced particle motion [3]), energy filters contribute to image enhancement by removing inelastically scattered electrons that contribute to background noise [4], while constant-power condenser lenses allow the switches to be made in optical settings necessary during high-resolution low-dose imaging protocols without affecting beam stability. In combination with advanced image processing algorithms [5–9] such a highly sophisticated set-up is capable of achieving resolutions between 3.0 Å to 2.5 Å, with a few reconstructions surpassing 1.8 Å resolution. Interestingly, most of the reconstructions resolved at a resolution of 2 Å or better correspond to apoferritin from various organisms (Fig 1D, Fig 1E, S1 Table), as a result of its high symmetry and intrinsic stability. Indeed, apoferritin is now commonly used as a standard sample to assess microscope performance and implement developments in cryo-EM [8, 10–13].

High resolution cryo-EM studies are therefore most likely to succeed for stable specimens imaged using high kV electron microscopes with energy filters and direct detection technology, in combination with modern image processing routines [14]. Such sophisticated equipment is however expensive, requiring high-level strategies to maintain microscope stability and performance, so that the method would appear to be inaccessible on a daily basis to many laboratories. Recent publications have explored the potential of 200 kV microscopes to obtain reconstructions higher than 3 Å [15–19], see S2 Table. With the exception of one group that uses a JEOL cryo-ARM^®^ microscope with an in-column energy filter and a sophisticated projection system, data for each of these have been collected using instruments of the Arctica^®^ microscope series, which includes a two-lens condenser system. Such dedicated instruments are costly and have a rather large footprint. These set-ups, equipped predominantly with Gatan K2^®^ or FEI Falcon^®^ DDD cameras, require dedicated space for their components. Interestingly, all maps higher than 2.8 Å have been resolved with the Gatan K2^®^ DDD, which has been used to resolve more than half (55%) of high-resolution electron density maps in the EMDB (Fig 1C). Finally, two of the highest resolution structures currently deposited at 200 kV use either an in-column or post-column energy filter.

Why are 200 keV microscopes generally considered to be sub-optimal for high resolution studies? Apart from the reduction in the high angle diffraction resolution limit at lower energy, the probability of electrons being inelastically scattered increases with decreased keV. This leads to an increase in the background noise of the acquired micrographs, as well as increased interactions with the sample, which in turn increases the likelihood of electron-induced radiation damage [20]. Inelastic scattering can be compensated for by the use of an energy filter, as demonstrated recently for mouse apoferritin [10], although installation costs can be prohibitive. On the other hand, 200 keV instruments offer distinct advantages for cryo-EM applications, including the higher contrast of the specimen [21], [22] and considerably lower maintenance requirements due to the simpler design.

Below, we describe our electron optical settings and protocols for high-resolution cryo-EM of apoferritin. We show that basic settings for automated cryo-electron microscopy imaging using the Glacios^®^ electron microscope, often thought of as a “screening microscope”, allow structure determination at high resolution. We use mouse H-chain apoferritin to produce an atomic model resolved at 2.7 Å and compare it to the corresponding 2.24 Å crystal structure (PDB ID: 3WNW [23]).

## Materials and methods

### Sample preparation and grid screening

A 3μl aliquot sample of 4.0 mg/ml concentration of mouse H-chain apoferritin was kindly provided by Thermo Fisher Scientific and Christos Savva (Leicester Institute of Structural and Chemical Biology, Leicester, UK). The sample was applied to glow-discharged Quantifoil holey carbon grids (R1.2/1.3, 200 mesh). Cryo-EM grids were prepared using Vitrobot Mark IV System (Thermo Fisher Scientific) with 95% humidity, ashless filter paper (Standard Vitrobot Filter Paper, Ø55/20mm, Grade 595) and blotting time of 4 sec.

### Cryo-EM data collection

Grids were imaged using a 200 keV Thermo Scientific Glacios^®^ Cryo-Transmission Electron Microscope (focal length 3.4 mm, C_*s*_ = 2.7 mm, objective aperture 100 μm) equipped with an X-FEG source. Humidity and temperature of the microscope environment were controlled with a regular, commercially available air-conditioning system, and DC and AC magnetic fields of the room were actively cancelled with a dedicated magnetic field cancellation system (MR-3 from Stefan Mayer Instruments), installed by MD-ECS (http://md-ecs.com/). Measurements of AC fields before and after installation are shown in the S2 Fig. Images were recorded using a Falcon IIIEC direct electron detector in counting mode. The images were collected using EPU software 2.2.0.65REL with an under focus range from 0.6 μm to 1.6 μm.

### Microscope settings (for further details, see S1 File)

Standard illumination alignments were performed prior to data collection. The gun was centered and tilted to have a central on-axis beam of electrons. The extracting voltage was set to the minimum of 4.2 keV to achieve a concentrated beam of low energy spread [24]. The strength of the first condenser lens (C1) was adjusted to achieve the desired dose rate of 0.93 electrons per pixel per second (e-/pix/sec). The second condenser lens (C2) was used for parallel beam illumination, achieved by optimizing the sharpness of the objective aperture and simultaneously adjusting the intensity of the electron beam to the highest possible illumination in diffraction mode. The sample for this adjustment was amorphous carbon areas of the Quantifoil grid, imaged at eucentric height and defocus of -1 μm. The C2 aperture was set to 50 μm as beam defining aperture to minimize the beam diameter corresponding to the field of view, yielding a beam diameter of ~1.7 μm. Astigmatism of the C2 lens was corrected. The objective lens complex was also precisely aligned. A 100 μm aperture was symmetrically set in the focal plane of the objective lens for limiting high-angle inelastic or multiple scattered electrons.

We performed a Young’s fringe test at the pixel size used for the subsequent acquisition of apoferritin to estimate information limit. The fringes extend to the border of the image, demonstrating that the information limit is near the Nyquist frequency of the image, i.e. 2*0.96Å = 1.92Å (see Conclusions section).

Objective astigmatism and coma were minimised iteratively using the autostigmate and autocoma routines in EPU on the amorphous carbon region of the grid. 300 4KX4K movies were collected with a Falcon IIIEC direct electron detector in counting mode, using EPU for automated data collection. The pixel size was set to 0.96 Å. Exposure was set to ~1 e-/Å^2^/sec and 30 frames were collected in total, with an overall dose of 28 e-/Å^2^. Applied defocus varied from -0.8 μm to -1.6 μm. Monitoring of image properties, estimated defocus and resolution was performed on-the-fly with the WARP software [25].

### Image processing and map calculation

Movies were imported and analyzed in Relion 3.0.5. and motion correction was performed by default. To calculate the CTF, gctf was employed with standard parameters. We initially used 1 CTF-corrected micrograph to manually pick 113 particles with the box size set to 256 pixels. Iterative 2D classification was performed with an applied circular mask of 200 Å. In the first iteration of 2D, using the 113 particles, the most populated class was selected for template-based particle picking, low-pass filtered to 20 Å. In a test set of 7 micrographs, 5,059 particles were picked; visual inspection showed that > 80% of particles were captured per micrograph. A second iteration of 2D classification was performed. The top 2 classes including 76% of the particles were again selected for template-based particle picking using default parameters. In all, 211,177 particles were auto-picked from 300 micrographs, and a third final iteration of 2D classification discarded 312 single particles, resulting in 210,865 individual particles. 3D classification in 3 classes was performed with an applied circular mask of 140 Å. We used as reference the 1.65 Å human apoferritin map resolved with Relion 3.0 (EMDB 0144), low-pass filtered to 30 Å. Octahedral O symmetry was applied. Resulting classes reached < 6.1 Å resolution, with the best and most populated class reaching 4.5 Å (45% of particles). Subsequent refinement of the best class reached an initial resolution of 3.6 Å. After 2 iterations of particle-based CTF refinement and particle polishing implemented in Relion 3.0, the final reconstruction of mouse apoferritin included 95,733 single particles and reached 2.73 Å resolution (FSC = 0.143).

### Model building and refinement

One protein monomer of the 2.24 Å resolution X-ray crystal structure of mouse H-chain apoferritin (PDB code 3WNW [23]) was readily placed into the octahedrally averaged cryo-EM Coulomb potential map ρ_map_ as a rigid body using Chimera [26] and the polypeptide chain fitted in real space using Coot [27].

**Fig 2:**
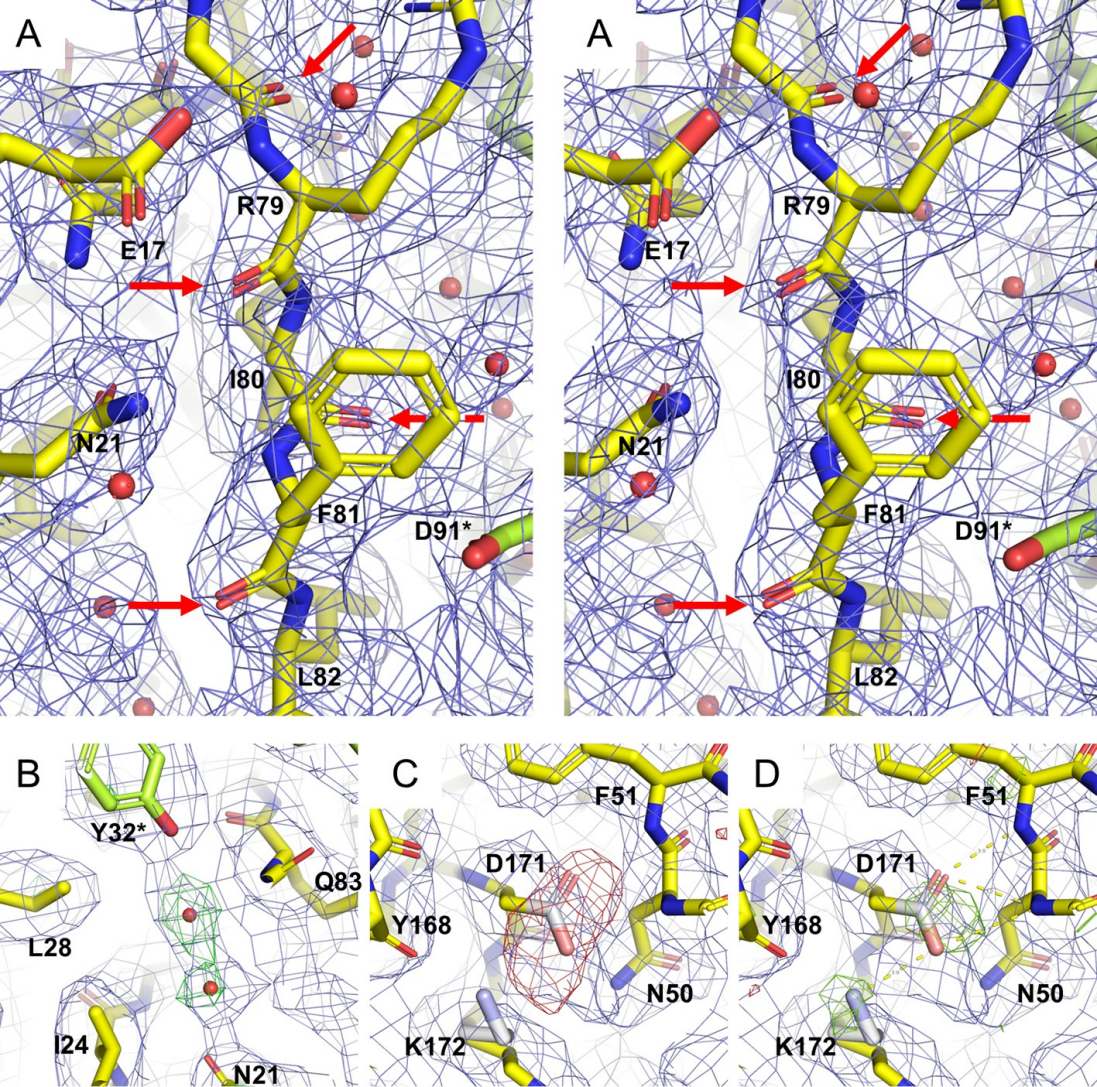
Representative densities during quasi-crystallographic refinement against the cryo-EM Coulomb potential map. (A) Stereo view demonstrating cryo-EM Coulomb potential map density (blue cage) for carbonyl oxygens (red arrows) in accordance with a nominal resolution of 2.7 Å. The final refined model (PDB code 6SHT) is shown as yellow sticks, with octahedrally symmetry related residues colored lime. (B) Positive difference electron density (F_map_-F_calc_), green, contoured at 2.5σ) after first round of quasi-crystallographic refinement of manually fitted apoferritin monomer superimposed with the final model (yellow / lime sticks). As the initial refinement was based on protein residues alone, the difference density clearly demonstrates the presence of two solvent molecules, also present in the cryo-EM map. These water molecules are also present in the crystal structure (PDB code 3WNW). (C) The initial refinement also exhibited negative difference density (red cage, contoured at -3.5σ) for all carboxylate groups (here Asp171). These atoms (Asp: C^γ^, O^δ1^ and O^δ2^: Glu: C^δ^, O^ε1^ and O^ε1^) were given an occupancy of zero so that they no longer contribute to structure factor calculations (i.e. set dummy, white / pink atoms). For a number of carboxylate groups including Asp171, the next round of refinement revealed positive difference density (D, green cage contoured at 3.5σ), demonstrating an influence of these atoms on the cryo-EM Coulomb potential. This round of refinement also revealed density for a second conformation of the Lys172 side chain (white sticks).

In order to maximize the agreement between the model and the map, we adopted a quasi-crystallographic approach. As the cryo-EM reconstruction ρ_map_ possesses octahedral (point group 432) symmetry, all symmetry elements of the apoferritin nanocage (and the map) are recapitulated if one monomer (the asymmetric unit) is suitably positioned (with respect to origin and symmetry axes) in the cubic space group P432, resulting in a significant reduction in computing requirements. The following procedure was followed (see Fig 2A–2D):

- ρ_map_ was converted to structure factors F_map_(hkl) using the function mrc2mtz from CCP-EM software suite [28] in the space group P432 with cell constants a = b = c = 245.76 Å (to ensure isolation of a single apoferritin nanocage)
- coordinates of the apoferritin monomer (asymmetric unit of the same “cell”) were refined in reciprocal space against the F_map_ data set in Phenix [29] using electron scattering factors
- refined model coordinates were displayed in Coot together with ρ_map_ and the F_map_-F_calc_ difference Fourier map. Superposition of positive difference density with ρ_map_ was used to guide placement of additional atoms (e.g. alternative side chain conformations and solvent molecules, Fig 2B), which were then fitted in real space against ρ_map_. Negative difference density highlighted misplaced atoms in the model (in the first round primarily carboxylate groups, Fig 2C); these were either repositioned as appropriate or set dummy (occupancy set to zero)
- the resulting coordinates were again subjected to reciprocal space refinement and the procedure repeated. Dummy carboxylate groups that showed positive difference density (e.g. Fig 2D) were reintroduced to the model.

This procedure was repeated until no further significant features appeared in the difference densities.

## Results and discussion

The electron microscope used here is equipped with an automated loading mechanism that minimizes manual intervention during the loading procedure and hence ice contamination. The illumination system includes a stable high-brightness Field Emission Source (X-FEG) and two basic condenser electromagnetic lenses, which lowers the cost of the system. Care must be taken to adjust and maintain beam parallelism, however. The objective lens is a constant power symmetric twin lens equipped with an auxiliary lens (the micro-condenser lens), which provides a narrow parallel beam for localization of the emission in the field of interest while avoiding any damage to the surrounding area.

Three main features contribute to reduced purchase, installation and maintenance costs of our setup (S1 and S2 Tables). The Glacios^®^ microscope column is closer to the ground, providing increased mechanical stability and reduced sensitivity to external vibrations (although this complicates the fitting of either an in-column or post-column energy filter). It also exhibits a reduced footprint that allows installation in standard laboratory rooms, resulting in increased accessibility and reduced infrastructure costs to control environmental conditions (including humidity, temperature, noise and field cancellation, see S2 Fig) that are critical for microscope stability [30]. Finally, the Falcon^®^ IIIEC DDD comes at a favorable price compared to other DDD detectors, at the expense of lower frame rates.

Lower frame rates can however present various drawbacks to high-resolution imaging, including: (a) longer exposure times, especially in counting mode, reducing throughput of the microscope; (b) an inability to correct for large sample drifts in movie mode and (c) impracticability of super-resolution data collection due to the low beam current that must be applied at the sensor level to counteract earlier saturation. In the present study, the Falcon^®^ IIIEC was used in counting mode for imaging, with 40 frames per second (fps) of 4KX4K and an electron dose of 0.97 e-/Å^2^/s. Vitrification at high concentration (4 mg/ml) allowed imaging of a suitable number of particles (S3A Fig) on only 300 micrographs in less than 12 hours. Frames were motion corrected with built-in microscope software frame alignment routines followed by frame alignment procedures in Relion 3.0.5 [8]. Final aligned stacks show global motions ~0.3 Å on average (S3A Fig). Applied and calculated defocus values varied between -600 nm and -1,600 nm and showed high consistency when calculated with gctf [31] (S3B Fig), demonstrating the robustness of the defocus calculation. Defocus values close to the Scherzer defocus allowed us to perform contrast transfer function (CTF) fitting with high confidence using gctf [31] (S3A–S3C Fig). These protocols resulted in consistent recording of electron micrographs with calculated average quality of information as defined by gctf of ~3.2 Å (S3B Fig).

**Fig 3:**
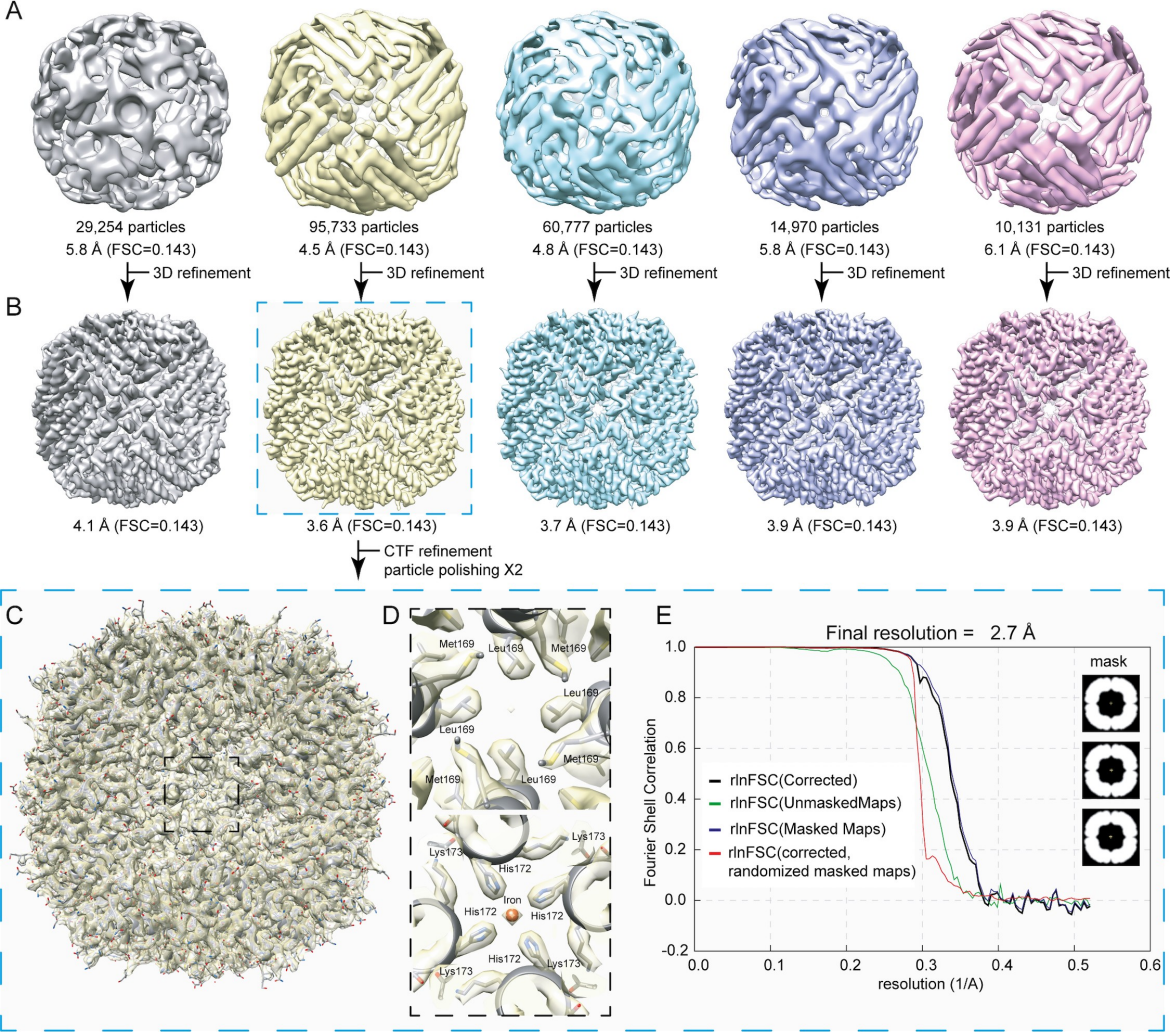
Image processing of single-particle data after 2D classification and final resolution estimation. (A) Five (5) 3D classes were initially calculated, reaching resolutions from 4.5 Å to 6.1 Å. (B) Subsequent 3D refinement procedures led to reconstructions of the classes in a resolution range of 3.6 Å to 4.1 Å. (C) Overlap of the final atomic model of mouse apoferritin with the refined Coulomb potential density map. (D) Close-up images along the 4-fold axis of apoferritin at different depths of the protein shell. Coulomb potential densities are recapitulated for the corresponding channel, including a bound iron atom. (E) Fourier shell correlation plot for the final 3D reconstruction shown in (C). At an FSC of a reported correlation value of 0.143, resolution reaches 2.7 Å.

Standard image processing procedures were performed in Relion 3.0.5 (Fig 3A–3E, S3 Fig) to calculate an electron optical density reconstruction of mouse apoferritin at 2.7 Å (Fig 3E). In particular, iterations of particle-based CTF refinement, followed by particle-based polishing and beam-induced motion correction procedures [9] were critical factors in increasing the resolution of the final reconstruction of mouse apoferritin from 3.6 Å to 2.7 Å (Fig 3E). To validate the final reconstruction, we calculated the FSC of unmasked, masked and phase randomized masks with Relion 3.0.5 (Fig 3E), and, in addition, evaluated the orientation distribution for single particles used for reconstructing the density using the efficiency measure (E_OD_) (S4A and S4B Fig) [32] and directional FSC metrics (S4C Fig) [33]. Overall, the efficiency calculated is high (E_OD_ = 0.8), demonstrating sufficiently uniform orientation distributions–validated by directional FSC plots with a sphericity of the 3D FSC of 0.992 (S4C Fig)–and comparing well with other published EM maps, which exhibit values between E_OD_ = 0.7 and E_OD_ = 0.9 [32]. We have also re-calculated the final EM map without symmetry, leading to a reconstruction of 3.1 Å (S5 Fig), showing that the same validity measures (E_OD_ = 0.8, sphericity = 0.990) are recapitulated for the asymmetric reconstruction (S5A–S5D Fig). Data collection and processing parameters are given in Table 1, and FSC plots comparing the cryo-EM final map and half-maps to the model are shown in S4 Fig. Recalculation of the cryo-EM map in the absence of symmetry (S5A–S5D Fig) confirmed that the observed apoferritin densities were not an artefact of symmetrization (S6 Fig).

**Table 1:** Cryo-EM data collection, refinement and validation statistics.

	*M*. *musculus* apoferritin (EMDB-10205) (PDB 6SHT)
**Data collection and processing**	
Magnification	150,000X
Voltage (kV)	200
Electron exposure (e–/Å^2^)	28
Defocus range (μm)	-0.8 to -1.6
Pixel size (Å)	0.96
Symmetry imposed	O
Initial particle images (no.)	211,177
Final particle images (no.)	95,733
Map resolution (Å)	2.73
FSC threshold	0.143
Map resolution range (Å)	2.73–3.21 (Relion)
	2.10–3.60 (ResMap)
**Refinement**	
Initial model used (PDB code)	3wnw
Model resolution (Å)	2.76
FSC threshold	FSC = 0.143
Model resolution range (Å)	2.76–2.99^a^
Map sharpening *B* factor (Å^2^)	-95.1618
Model composition	
Non-hydrogen atoms	1474
Protein residues	173
Ligands	74
*B* factors (Å^2^)	
Protein	34.8
Ligand	36.6
R.m.s. deviations	
Bond lengths (Å)	0.015
Bond angles (°)	1.524
Validation	
Clashscore	5.96
Poor rotamers (%)	2.5
Ramachandran plot	
Favored (%)	98.9
Allowed (%)	0.5
Disallowed (%)	0.6

^a^resolution range includes resolution values reported at FSC = 0.143 against half-maps, unprocessed, unmasked map and the final processed masked map (S4 Fig).

**Fig 4:**
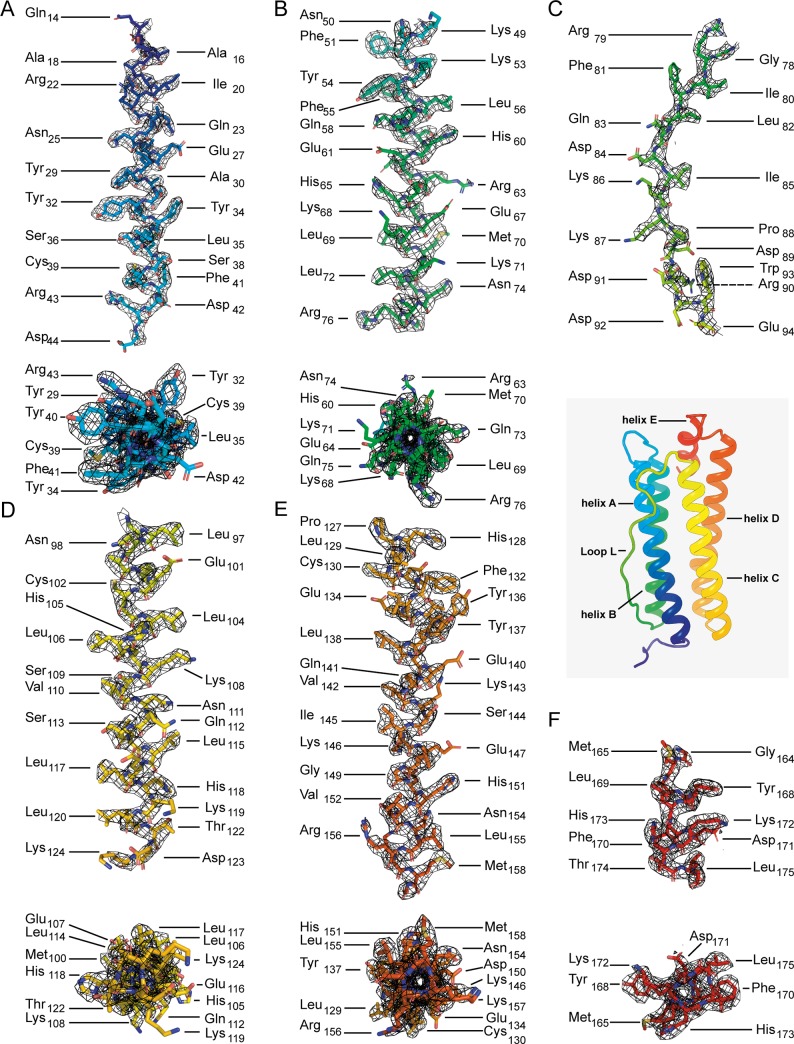
Coulomb potential densities for each secondary structure element of the apoferritin monomer (insert), with individual amino acid residues shown in stick representation. (A, B, D-F) helices A-E are shown with the fitted model and the corresponding density perpendicular to (top panels) and along (bottom panels) the helix axes. (C) Density and fitted model for loop L.

Overall, the architecture of mouse apoferritin is as expected (Fig 3C), and two layers along the 4-fold channel axis in which an iron atom is bound is illustrated along with the resolved densities in Fig 3D. The Coulomb potential map clearly resolves densities for all secondary structure elements of apoferritin (helices A-E and Loop L (Fig 4)), with the appearance of main chain carbonyl oxygens (Fig 2A) consistent with the FSC-calculated resolution.

**Fig 5:**
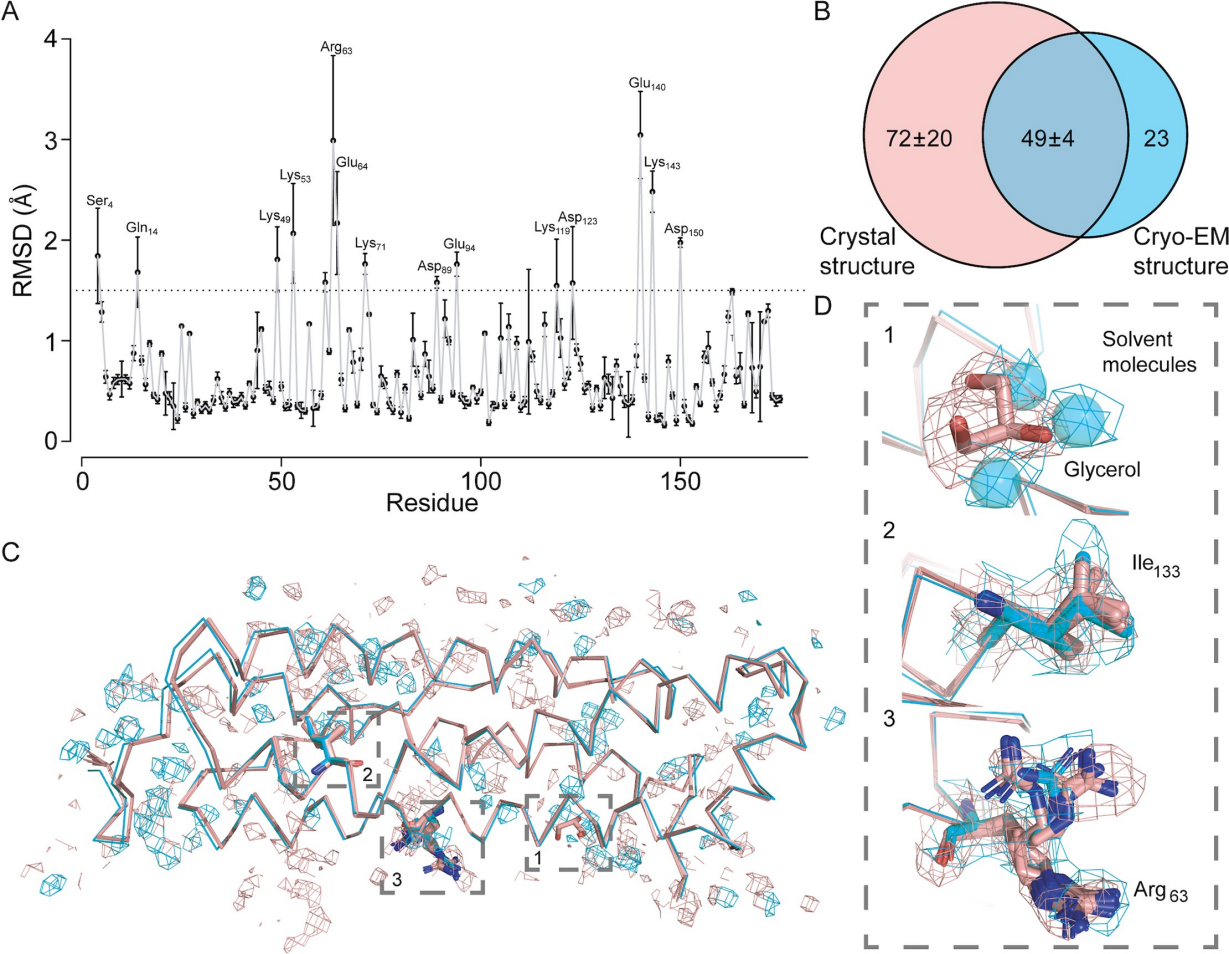
Comparison of the cryo-EM-derived structure to its crystallographic counterpart. Note that there are 12 monomers in the crystallographic asymmetric unit, so that comparisons are shown against all. (A) per-residue side-chain root-mean-square deviation (RMSD, Å) for all atoms; backbone atoms show an overall RMSD of 0.395 Å. Residues exhibiting RMSD values > 1.5 Å are highlighted in the plot. (B) Venn diagram showing overlap of solvent molecules derived from the cryo-EM map with those of the crystallographic monomers. (C) overlay of Cα atoms from the cryo-EM model (cyan) and the crystallographic monomers (pink) together with corresponding densities for the solvent. Boxes 1–3 denote positions shown in (D). (D) Comparison of cryo-EM (cyan) and X-ray (pink) models for selected residues with corresponding density/densities.

Following quasi-crystallographic model refinement, the final coordinate set (PDB deposition 6sht) consists of 1474 protein atoms (173 residues), two putative cations (Fe^2+/3+^ and Mg^2+^) and 72 solvent atoms with a quasi-crystallographic R_work_/R_free_ of 0.2509/0.2543 (see Table 1). The final model is in good agreement with the 2.24 Å X-ray crystallographic structure 3WNW, with a backbone RMSD of 0.395 Å (Fig 5). While the structures display the same overall main chain conformation, side chain rotamers of a few amino acids (in particular charged side chains) display a broader distribution (Fig 5A). This is likely due to their predominant surface location, leading (in both crystal and cryo-EM structures), to statistical / dynamic fluctuations in solvent exposed regions [34], although crystal packing and electron induced radiation damage may also play a role.

Side chains are generally well resolved, with the unambiguous observation of *cis*Pro161 underlining the quality of the reconstruction (S7 Fig). Similarly, polar (e.g. Asn98, Gln112) and aromatic (e.g. Trp93) residues are well resolved, independent of contour level. On the other hand, densities for most acidic side chain carboxylates are observed at low contour levels, *e*.*g*. for Asp131 and Glu134 (S7 Fig). It is often argued that such reduced/absent density is due to the enhanced sensitivity of acidic side chains to electron radiation and subsequent damage [35, 36] which is more prominent at 200 keV compared to apoferritin structures resolved at 300 keV [8, 11] due to increased inelastic scattering. Our difference Fourier techniques however demonstrate that these atoms are present in our maps, at least for some carboxylate groups (Fig 2D). The negative charge at carboxylic acid atoms has a profound effect on the elastic electron scattering factor [37, 38], resulting in depression of the Coulomb potential map value [39–43].

Multiple side chain conformations could be modelled (e.g. Lys172, Fig 2D and Ile133, Fig 5), as commonly found in X-ray crystallographic structures. The side chain of Arg63, a residue at a two-fold symmetry interface between two protomers, could be built in two orientations (Fig 5). In the tetragonal crystal form (pdb id 3WNW), which has twelve independent monomers in the asymmetric unit, this side chain interacts alternately with acidic side chains Glu64 and Glu67 of the same monomer and Glu64* and Glu67* of the opposing monomer depending on the location in the nanocage. Thus, the two conformations seen in the cryo-EM map simultaneously recapitulate the crystallographic structure but are also a result of the octahedral averaging.

The 2.7 Å 200 kV cryo-EM potential map also revealed density for 72 solvent molecules, compared to an average of 121 +/- 24 water molecules per monomer of the asymmetric unit in the 2.24 Å X-ray crystallographic structure (Fig 5B). Of these, 49 +/- 4 (68.1%) of the positions overlap, demonstrating the congruence of the cryo-EM and X-ray crystallographic structures and pointing to a structural role for these solvent molecules. Notably, two glycerol molecules per monomer observed in the crystal structure (from the cryo buffer) are replaced by solvent molecules in our structure (Fig 5C and 5D). Two putative cations could also be located (although their identification cannot be determined from the present cryo-EM map alone): an iron ion on the fourfold symmetry axis [44] (Fig 3D) and a magnesium ion at the ferroxidase site [45] of the monomer (S8A Fig). The crystal structure also shows two further magnesium ions arranged off-axis around the local three-fold axis together with coordinating solvent molecules. Although the cryo-EM density is consistent with a three-fold averaging of these (S8B Fig), these were not included in the model due to said averaging. In contrast, no cryo-EM density was present for a potassium ion in the crystal structure.

Application of our imaging protocols to mouse apoferritin thus demonstrates that our cryo-EM model recapitulates its X-ray crystallographic counterpart. The overall fold, main chain organization, side chain and solvent molecule densities are readily interpretable from the reconstruction. In particular, functional channels of the molecule are well resolved. Nevertheless, the densities for negatively charged amino acid residues are consistently lower in the cryo-EM map, a phenomenon that is likely to be more prominent in 200 keV than in 300 keV reconstructions due to increased inelastic scattering. In addition, negatively charged atoms result in attenuated positive potentials and corresponding charge density maps [39–43]. Our observations are consistent with previously resolved cryo-EM maps derived from 200 keV electron microscopes [17, 18].

## Conclusions

Our quasi-crystallographic approach offers some distinct advantages for model building in cryo-EM maps. If parameterized correctly, the Fourier transformation remains a faithful representation of the cryo-EM map. Alternation between real and reciprocal space brings with it the benefit that any local errors in the map are dispersed throughout reciprocal space and *vice versa*. Well-established crystallographic routines can be used to refine the model against the experimental map. Most importantly, difference Fourier maps allow identification of missing atoms or misinterpreted parts of the model, providing a reliable guide to extracting the maximum information from the experimental map to the model coordinates. In particular, our results show that even in our cryo-EM map at 2.7 Å resolution, information concerning solvent molecules and charge states can be discerned. Electron scattering factors for ionized atoms have been parametrized [37, 38] and first applications to electron crystallographic studies and cryo-EM single particle reconstructions have been reported [46]. Development of protocols to refine atomic (partial) charge states and their application to high-resolution cryo-EM single particle reconstructions would realize the full potential of cryo-EM in excess of “simple structure determination” [39–43].

The resolution of 2.7 Å achieved here is unlikely to be the limit–the Young’s fringe test from our Glacios^®^ microscope indicates an information limit of under 2 Å (S9 Fig). We expect that use of an energy filter would allow achievement of higher resolution, as demonstrated recently for a reconstruction of mouse apoferritin at 2.0 Å, where images were recorded with a higher frame rate detector and a more sophisticated 200 keV microscope [10]. Resolution could be further increased by benchmarking vitrification conditions to achieve even thinner vitreous ice, which will further reduce the noise. Higher concentrations of the sample, resulting in more particles per image, and in general, larger numbers of acquired micrographs containing increased numbers of single particles would also further increase resolution. In the present study, we selected a pixel size of our detector of 0.96 Å/pixel, which is around half the information limit of the optical system (S9 Fig). Capturing molecules at higher magnifications, resulting in smaller pixel sizes at the detector level, may also allow reconstruction at higher-resolution. Acquiring movies in super resolution model and/or use of direct electron detectors with competitive dynamic quantum efficiency (*e*.*g*. Gatan K2^®^ or Gatan K3^®^) would further reduce noise and thereby increase the resolution of the Coulomb potential maps.

Software improvements can also contribute, especially those that take optical aberrations and anisotropic magnifications into account. For example, RELION 3.1 can, in principle, estimate and consider higher-order aberrations and anisotropic magnification *a posteriori*, improving the resolution of the final reconstruction, although attention must be paid in the use of Zernike polynomials to model higher-order aberration effects [47]. Small tweaks in image processing parameters, including benchmarking voltage, values of spherical aberration (C*s*) and further optimization of per-particle CTF refinement, can potentially lead to higher resolution reconstructions. It is of note that the Lander laboratory have recently communicated resolutions for apoferritin better than 2.0 Å at 200 kV by considering various of the above-mentioned factors on an Arctica^®^ microscope with a Gatan K2^®^ electron detector [48].

Overall, we expect that similar resolution can be achieved using the Glacios^®^ on the condition that the sample and the vitrification process are optimal and the above-mentioned factors are taken into consideration. Naturally, this resolution will be harder to achieve for samples with increased heterogeneity or ice thickness. Nevertheless, we have demonstrated that our electron optical settings and image processing methods allow structure determination of vitrified biological macromolecules at 2.7 Å with the Glacios^®^, providing an affordable option for in-house high-resolution structural biology.

## Supporting information

S1 File (DOCX)

S1 Fig Matrix highlighting the statistics of point group symmetries of cryo-EM maps solved at resolutions better than 3.0 Å. The whole square shows the 275 structures, and the size of the different boxes highlights the structures resolved with the corresponding symmetry; each symmetry type is indicated with its corresponding color, as shown in both the matrix and the insert. 31 of the C1 (asymmetric) reconstructions correspond to those of ribosomes.
(DOCX)

S2 Fig Effect of active field cancelling system on external magnetic fields. Measurements were performed at FEG height, demonstrating that the new installation is within specifications in regards to A/C (left panel) and D/C (right panel) fields. Indications a-f show switching off (a, c and e) and on (b, d, and f) the field cancellation system. External fields are effectively cancelled in all directions below required specifications set for the microscope, shown with grey and black horizontal dotted lines.
(DOCX)

S3 Fig Statistics of acquired micrographs and initial 2D classification of apoferritin particles. (A) Left: a typical cryo-EM micrograph with recognizable apoferritin particles at a concentration of 4.0 mg/ml. Right: double-corrected motion corrected values are shown for the 300 acquired images as a bar plot. Bottom right: a series of calculated defocus values for the acquired micrographs is shown that recapitulate the applied defocus values determined for the data acquisition. (B) A bean plot showing the distribution of calculated micrograph information quality derived from gctf fit; the black line shows the median; white lines represent individual data points; grey polygons represent the estimated density of the data. (C) 2D classification procedure to collect single-particles from all micrographs and subsequently select the 211,177 particles that were classified in five (5) classes in 3D (Fig 2).
(DOCX)

S4 Fig **Validation metrics for apoferritin using cryoEF (A-B), 3DFSC (C) and FSC model vs maps (D-E).** (A) Boxplot showing the distribution of 824 calculated point spread function (PSF) resolution; the cross marks the average, whereas the black bar shows the median. (B) Percentages of PSF resolution in different ranges (a-j). (C) Histogram and directional FSC plot as calculated by 3DFSC. (D) Fourier Shell correlation of derived apoferritin model after generation of symmetry mates at 100 Å and low-pass filtering the map to 2.7 Å, against (1) half-map 1, (2) half-map 2; (3) unprocessed map and (4) final map. Blue line corresponds to FSC of model vs the corresponding asymmetric cryo-EM reconstruction and orange line to FSC of model vs the corresponding octahedrally-symmetrized cryo-EM reconstruction.
(DOCX)

S5 Fig Validation metrics for apoferritin reconstructed without symmetry. **(**A) Fourier shell correlation plot for the derived 3D reconstruction. At an FSC of a reported correlation value of 0.143, resolution reaches 3.1 Å. (B) Boxplot showing the distribution of 836 calculated point spread function (PSF) resolution; the cross marks the average, whereas the black bar shows the median. (C) Percentages of PSF resolution in different ranges (a-j). (D) Histogram and directional FSC plot as calculated by 3DFSC. (E) Fourier Shell correlation of derived apoferritin model against (a) full map; (b) half-map (1) and (c) half-map (2); (d) refined against one half-map and correlated against the other.
(DOCX)

S6 Fig Assessment of observed density in reconstruction of apoferritin using different symmetry. (A) Model of apoferritin in the octahedrally-reconstructed cryo-EM map, highlighting tunnel residues and densities for bound elements. (B) Recovery of bound elements of the apoferritin map, using C1 symmetry. In grey color, the symmetrized cryo-EM map is depicted; in blue, the unsymmetrized map is shown, using the same single particles. Densities for bound elements are still visible. (C) Model of apoferritin superimposed in the unsymmetrized cryo-EM map, highlighting tunnel residues and densities for bound elements. An iron cation (orange), previously found in apoferritin structures, is highlighted.
(DOCX)

S7 Fig Side chains of helix E at different contour levels of the EM density (see text for details). (DOCX)

S8 Fig Apoferritin metal ion (grey transparent spheres) binding sites in our cryo-EM reconstruction (cyan) overlaid with the crystal structure (pink). (A) ferroxidase site (Mg^2+^), (B) three-fold axis channel (Mg^2+^) and (C) four-fold axis channel (Fe^2+^/Fe^3+^).
(DOCX)

S9 Fig Young’s fringe on a combined test specimen to estimate the information limit of the microscope at 150kX magnification, corresponding to pixel size of 0.96 Å; Objective aperture was set to 100μm and defocus to -0.1 μm. Briefly, a combined test specimen was used at the defocus -0.1. An image in pixel size of 0.96 was taken from the sample (S9A Fig) then the sample was slightly moved and another image at the same magnification was taken (S9B Fig). Two images were then summed pixel by pixel (S9C Fig) and the FFT of the new summed image were calculated by Thermo Fisher Scientific TIA software (S9D Fig). (A-B) Images of the combined specimen of the same region, with a slight shift of the stage; (C) Overlay of the two images (A and B) by pixel addition; (D) FFT of image (C), showing extension of the fringes to the border of the pattern.
(DOCX)

S1 Table Cryo-EM reconstructions resolved at 200 kV < 3.0 Å. The data set described in this manuscript is highlighted in blue.
(DOCX)

S2 Table Reconstructions of apoferritin resolved at resolutions < 3.0 Å. The data set described in this manuscript is highlighted in blue.
(DOCX)

S3 Table Optical presets used in this manuscript (see S1 File). (DOCX)
