# Supplementary material for: 2.7 Å cryo-EM structure of vitrified M. musculus H-chain apoferritin from a compact 200 keV cryo-microscope
Source: PLoS One. 2020 May 6;15(5):e0232540. doi: 10.1371/journal.pone.0232540 (PMC7202636; doi:10.1371/journal.pone.0232540)
Supplement: S1 File — (DOCX) [file pone.0232540.s001.docx]

**Supplementary Materials and Methods**

*Microscope alignments.* The Thermo Fisher Glacios^®^ Field Emission Cryo-Transmission Electron Microscope (Glacios^®^), a conventional TEM equipped with X-FEG Schottky Emitter, was used for the present studies. The electron gun of the electron microscope was set at an operating potential difference of 200 kV, extraction voltage of +4100 V and gun lens number 4. In this situation, the gun emission current was 272 μA at the time of the experiment. Alignments were performed as follows:

1. Column alignments. The column of the microscope was aligned using the standard complete column alignment procedure of the Talos/Glacios^®^ software. These alignments include gun alignment, nanoprobe (NP) alignment, high (HM) and low (LM) magnification TEM alignments and astigmatism corrections. The user is directed by the software to perform these alignments stepwise and are comprehensively described elsewhere (Talos on-line help manual -- Alignments, pp 1-83: Thermo Fisher Scientific, 2018). The goal of the gun alignment procedure is to have the most intense and symmetric illumination around the optical axis and to remove beam movement during changing C1 (i.e. spot size).  The NP, HM and LM alignments ensure the alignment of the optical axis and the centeredness and symmetry of the electron beam around the optical axis. We aimed at minimizing the changes in illumination and image formation conditions on changing spot size, magnification and microprobe-to-nanoprobe beam modes. Finally, astigmatism corrections include optimizing the symmetry of C2, objective and diffraction lenses to achieve optimal circularity of beam shape, Thon rings and diffraction pattern, respectively.

Column alignment was performed several days before image acquisition, although our experience with the Glacios^®^ indicates that the column alignments are quite stable over a few months. Nevertheless, some check-ups and fine alignments are always necessary prior to high-resolution data acquisition, including beam shift, C2 and objective aperture centeredness as well as C2 and objective lens astigmatism and coma-free. The order of these alignments is described in detail in section 3 (Direct Alignments).

2. Workflow for imaging. After loading the sample into the column, a distinct feature at all working magnifications was selected manually to make rough corrections to the eucentric height (by minimizing movement of the feature during the stage tilt) as well as to remove image shifts at desired magnifications (i.e. image shift calibration). The latter was performed by bringing the feature to the center in all desired magnifications, recording the values of image shifts and applying these to each working magnification.

A low magnification atlas overview of the grids was prepared with the optical settings and microscope and camera parameters presented in Table S3 (Atlas). Images of each part of the grid were recorded together with the physical location of each pixel. The applied dose at this optical setting was negligible.

After evaluating the atlas, a single grid square with thin vitrified water and good distribution of particles was selected for further acquisition. The exact eucentric height (EH) of the center of this grid square was measured by beam tilt and cross-correlation of the beam tilt images using the microscope and camera settings in Table S3 (Hole/EH preset). An image of the grid square was then recorded in the Gridsquare setting (Table S3) at very low dose. The locations of the holes on the carbon film that contained thin vitrified water film were selected by the hole selection tool of EPU on the recorded image of the grid square; holes near the edges of the grid bars and those containing visible contamination were removed from the list manually. After hole selection, an acquisition template was defined for the software for the microscope to go through the following cycle:

a. Move stage to the saved positions of the holes, set the microscope and camera setting to Hole/EH preset (see Table S3).

b. Take an exposure at a pixel size of 0.85 nm to image the hole.

c. Center the image of the hole using a stage shift, wait for 30 s and take another image at the hole.

d. Move the image and the beam to the carbon area according to a predefined displacement vector, set the microscope and camera parameters to Autofocus (Table S3) and wait for 5 s.

e. Focus on the carbon using the objective lens current to the defocus value of -2 μm.

f. Move the image and the beam back to the center of the hole, set the defocus to the next value in the list of defoci using objective lens current, set the microscope and camera parameters to Data Acquisition preset (Table S3) and wait for 5 s.

g. Acquire image using the Data Acquisition preset.

3. Direct Alignments. After defining the template for the software and just before starting the automated data acquisition, the alignment of the microscope was checked and fine-tuned in the following order:

3.1 Beam shift

3.2 C2 Aperture Centering and C2 Astigmatism

3.3 Pivot Point Alignment

3.4 Parallel Beam Alignment

3.5 Objective Astigmatism

3.6 Coma-free (and objective current centering)

3.7 Objective Aperture Centering and

3.8 Fine Objective Astigmatism

Steps 3.1 to 3.3 were performed in an empty region of the sample (empty/destroyed carbon film) and in eucentric focus, while steps 3.4 to 3.8 were done on the stable carbon film.

*Steps 3.1-3.3.* The direct alignment procedure starts with a beam shift alignment in the data acquisition preset. For that purpose, after finding a broken empty region and setting the objective lens to the eucentric focus preset, the beam was converged using the C2 lens and the crossover was moved to the center of the screen using C2 beam deflectors. After centering the beam, the C2 aperture is also centered in a way that the beam converges and diverges concentrically while changing the C2 lens current, and the C2 astigmatism checked. After centering the beam, the beam tilt pivot point was corrected in both x and y directions in the image plane by balancing the currents of the first and second deflector sets to have no shift while tilting the beam. After these alignments, a beam shift alignment was performed again, as , the centeredness of the beam changes slightly as a result of pivot point alignment.

*Steps 3.4-3.8.* A stable carbon area in another, vicinal grid square was selected and brought into the beam for the further alignment steps. The eucentric height of the area was corrected using the Hole/EH pre-set and the beam tilt method, which minimizes the image movement in the different beam tilts. As this changes the elevation of the sample using the stage movement in Z direction, we fine-tuned the objective lens current to focus on the carbon area in the Autofocus pre-set using the beam tilt method. To obtain beam parallelism, the focus was set to the defocus value of -2 μm, the optical configuration changed from imaging to diffraction and an objective aperture of 100 μm was selected. The aperture was placed in the focal plane, the image of the aperture brought to focus using the diffraction lens, and the diffraction pattern focused using the C2 lens. After this adjustment, the microscope was returned to imaging mode and the value of the C2 lens selected for Data Acquisition, Autofocus and Thon Rings (Table S3). In our case, the C2 lens value was 37.905% and the beam diameter was 1.7μm with a C2 aperture size of 50 μm. The objective aperture was then retracted for further alignment steps.

The objective astigmatism was corrected using Thon ring ellipticity with the optical pre-set Thon Rings in EPU (Table S3). The same optical pre-set was used for coma-free alignment, where the Zemlin tableau method [1] was applied to achieve a symmetric beam tilt in all directions. Since the orientation of the beam changes slightly after this alignment, astigmatism aberration was again corrected for: after returning the 100μm objective aperture and centering, objective astigmatism was corrected by following Thon ring ellipticity.
