# Supplementary material for: 2.7 Å cryo-EM structure of vitrified M. musculus H-chain apoferritin from a compact 200 keV cryo-microscope
Source: PLoS One. 2020 May 6;15(5):e0232540. doi: 10.1371/journal.pone.0232540 (PMC7202636; doi:10.1371/journal.pone.0232540)
Supplement: S1 Fig — The whole square shows the 275 structures, and the size of the different boxes highlights the structures resolved with the corresponding symmetry; each symmetry type is indicated with its corresponding color, as shown in both the matrix and the insert. 31 of the C1 (asymmetric) reconstructions correspond to those of ribosomes. (DOCX) [file pone.0232540.s002.docx]

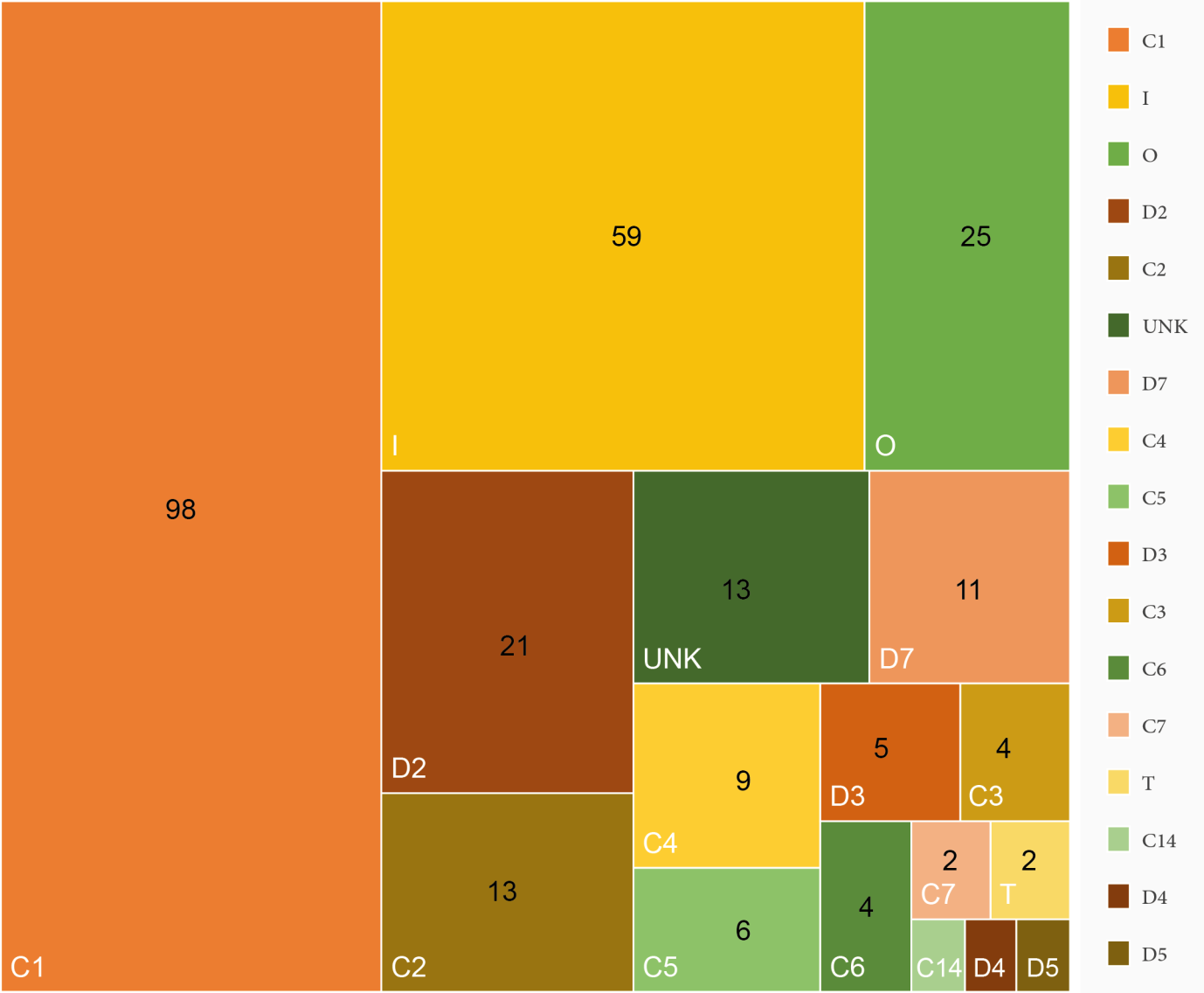
 **S1 Fig. Matrix highlighting the statistics of point group symmetries of cryo-EM maps solved at resolutions better than 3.0 Å.** The whole square shows the 275 structures, and the size of the different boxes highlights the structures resolved with the corresponding symmetry; each symmetry type is indicated with its corresponding color, as shown in both the matrix and the insert. 31 of the C1 (asymmetric) reconstructions correspond to those of ribosomes.
