# Supplementary material for: 2.7 Å cryo-EM structure of vitrified M. musculus H-chain apoferritin from a compact 200 keV cryo-microscope
Source: PLoS One. 2020 May 6;15(5):e0232540. doi: 10.1371/journal.pone.0232540 (PMC7202636; doi:10.1371/journal.pone.0232540)
Supplement: S2 Fig — Measurements were performed at FEG height, demonstrating that the new installation is within specifications in regards to A/C (left panel) and D/C (right panel) fields. Indications a-f show switching off (a, c and e) and on (b, d, and f) the field cancellation system. External fields are effectively cancelled in all directions below required specifications set for the microscope, shown with grey and black horizontal dotted lines. (DOCX) [file pone.0232540.s003.docx]

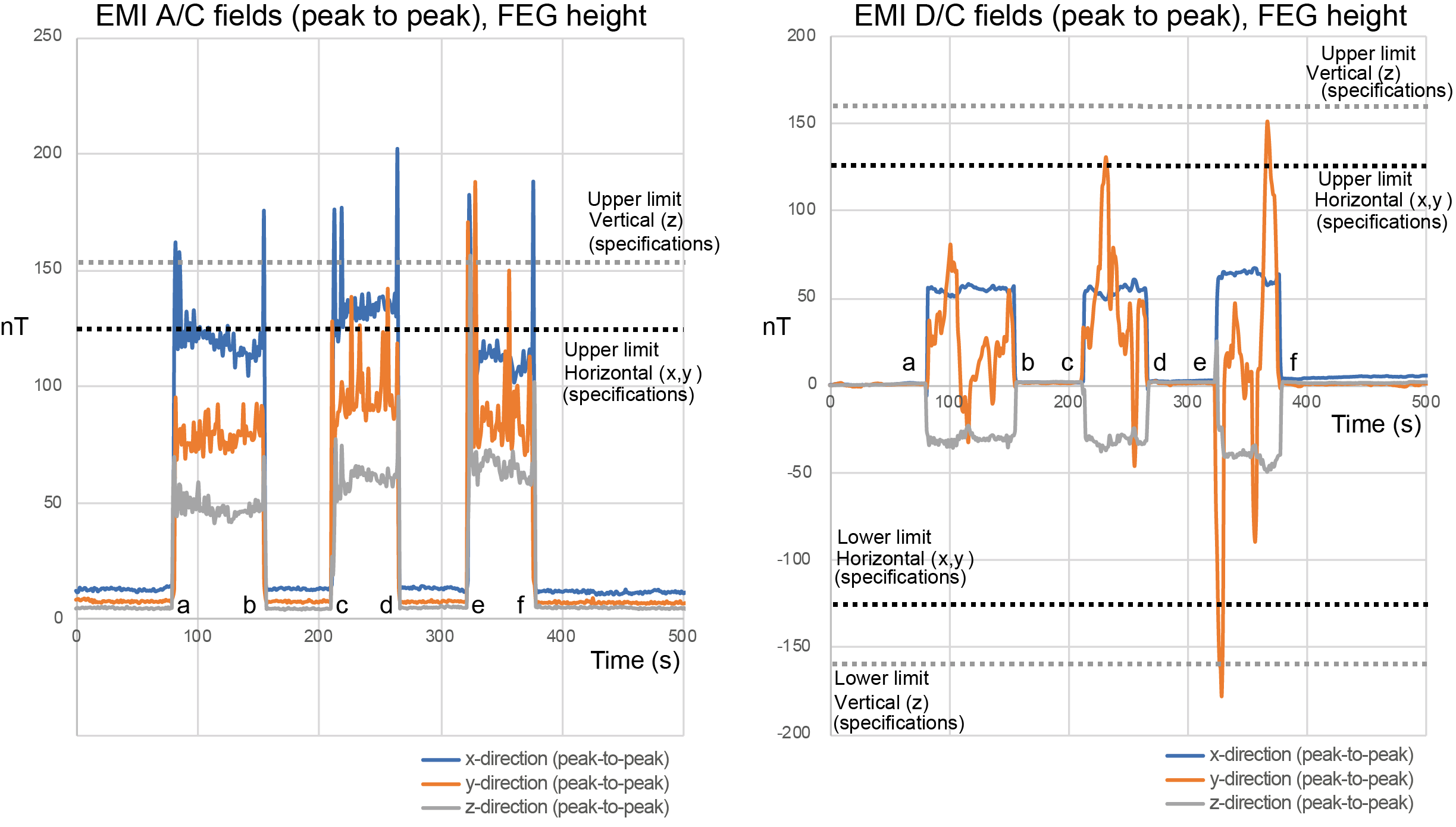


S2 Fig. Effect of active field cancelling system on external magnetic fields; Measurements were performed at FEG height, demonstrating that the new installation is within specifications in regards to A/C (left panel) and D/C (right panel) fields. Indications a-f show switching off (a, c and e) and on (b, d, and f) the field cancellation system. External fields are effectively cancelled in all directions below required specifications set for the microscope, shown with grey and black horizontal dotted lines.
