# Supplementary material for: 2.7 Å cryo-EM structure of vitrified M. musculus H-chain apoferritin from a compact 200 keV cryo-microscope
Source: PLoS One. 2020 May 6;15(5):e0232540. doi: 10.1371/journal.pone.0232540 (PMC7202636; doi:10.1371/journal.pone.0232540)
Supplement: S3 Fig — (A) Left: a typical cryo-EM micrograph with recognizable apoferritin particles at a concentration of 4.0 mg/ml. Right: double-corrected motion corrected values are shown for the 300 acquired images as a bar plot. Bottom right: a series of calculated defocus values for the acquired micrographs is shown that recapitulate the applied defocus values determined for the data acquisition. (B) A bean plot showing the distribution of calculated micrograph information quality derived from gctf fit; the black line shows the median; white lines represent individual data points; grey polygons represent the estimated density of the data. (C) 2D classification procedure to collect single-particles from all micrographs and subsequently select the 211,177 particles that were classified in five (5) classes in 3D (Fig 2). (DOCX) [file pone.0232540.s004.docx]

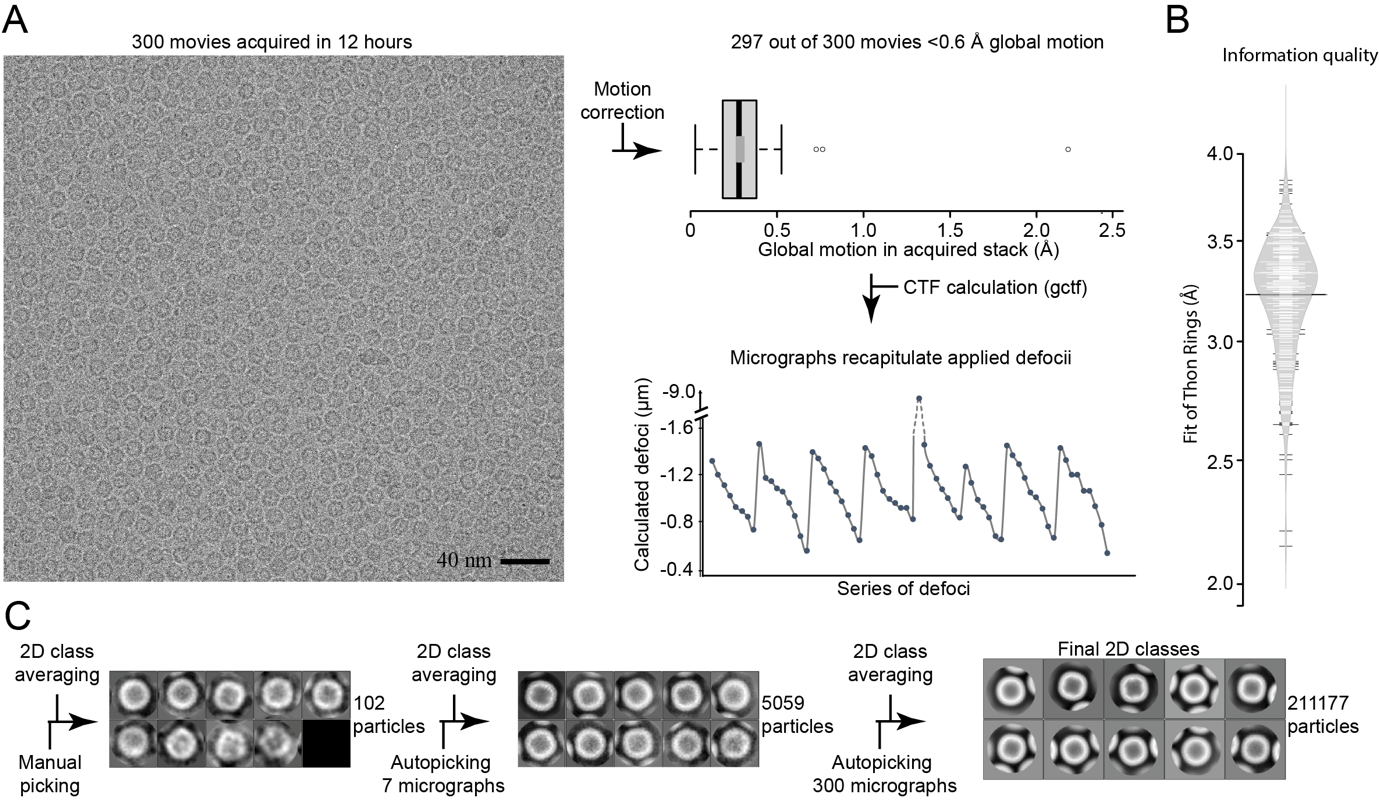


S3 Fig. Statistics of acquired micrographs and initial 2D classification of apoferritin particles. (A) Left: a typical cryo-EM micrograph with recognizable apoferritin particles at a concentration of 4.0 mg/ml. Right: double-corrected motion corrected values are shown for the 300 acquired images as a bar plot. Bottom right: a series of calculated defocus values for the acquired micrographs is shown that recapitulate the applied defocus values determined for the data acquisition. (B) A bean plot showing the distribution of calculated micrograph information quality derived from gctf fit; the black line shows the median; white lines represent individual data points; grey polygons represent the estimated density of the data. (C) 2D classification procedure to collect single-particles from all micrographs and subsequently select the 211,177 particles that were classified in five (5) classes in 3D (Fig 2).
