# Supplementary material for: 2.7 Å cryo-EM structure of vitrified M. musculus H-chain apoferritin from a compact 200 keV cryo-microscope
Source: PLoS One. 2020 May 6;15(5):e0232540. doi: 10.1371/journal.pone.0232540 (PMC7202636; doi:10.1371/journal.pone.0232540)
Supplement: S4 Fig — Validation metrics for apoferritin using cryoEF (A-B), 3DFSC (C) and FSC model vs maps (D-E). (A) Boxplot showing the distribution of 824 calculated point spread function (PSF) resolution; the cross marks the average, whereas the black bar shows the median. (B) Percentages of PSF resolution in different ranges (a-j). (C) Histogram and directional FSC plot as calculated by 3DFSC. (D) Fourier Shell correlation of derived apoferritin model after generation of symmetry mates at 100 Å and low-pass filtering the map to 2.7 Å, against (1) half-map 1, (2) half-map 2; (3) unprocessed map and (4) final map. Blue line corresponds to FSC of model vs the corresponding asymmetric cryo-EM reconstruction and orange line to FSC of model vs the corresponding octahedrally-symmetrized cryo-EM reconstruction. (DOCX) [file pone.0232540.s005.docx]

.
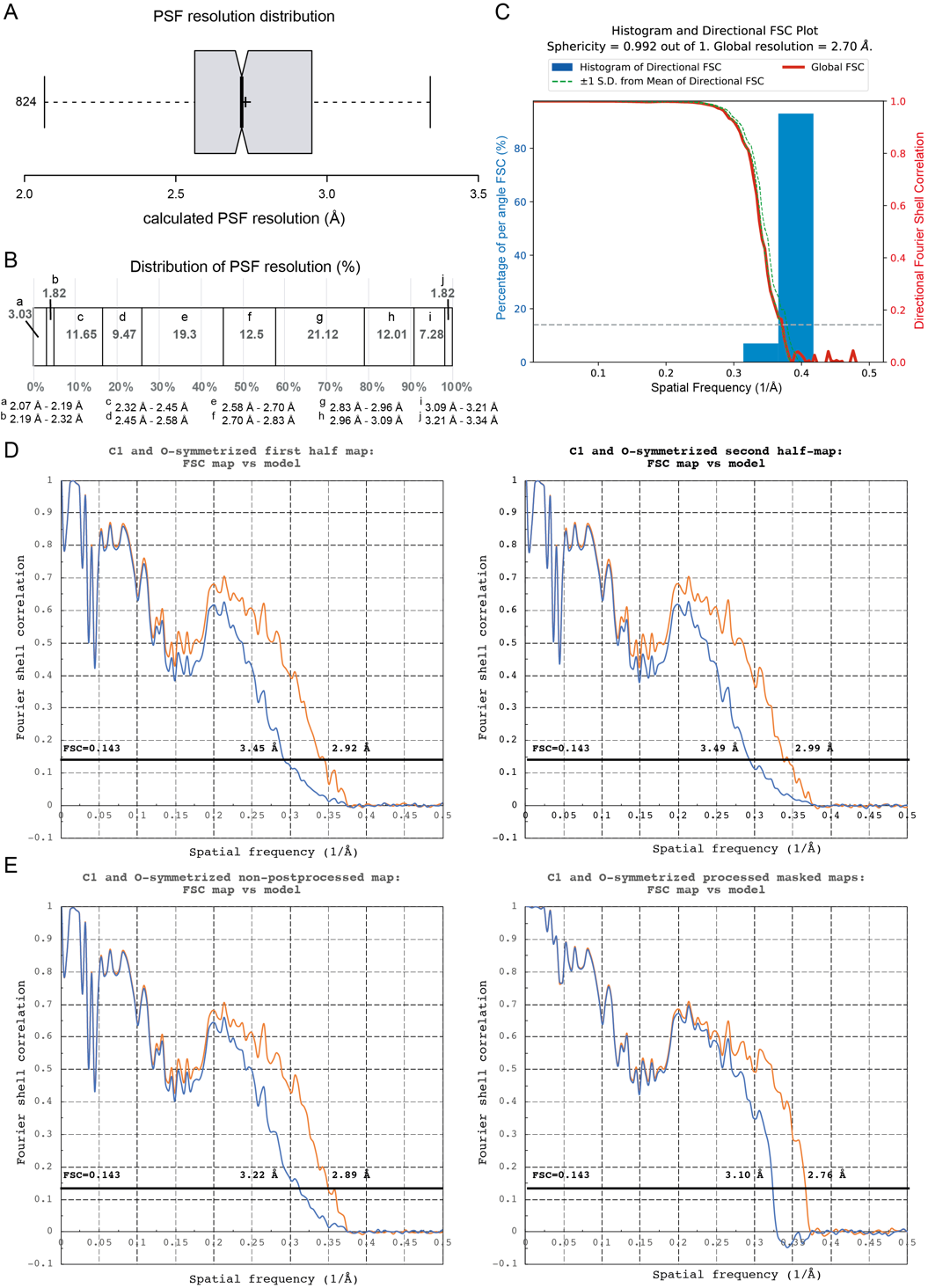


S4 Fig. Validation metrics for apoferritin using cryoEF [2] (A-B), 3DFSC [3] (C) and FSC model vs maps (D-E). (A) Boxplot showing the distribution of 824 calculated point spread function (PSF) resolution; the cross marks the average, whereas the black bar shows the median. (B) Percentages of PSF resolution in different ranges (a-j). (C) Histogram and directional FSC plot as calculated by 3DFSC. (D) Fourier Shell correlation of derived apoferritin model after generation of symmetry mates at 100 Å and low-pass filtering the map to 2.7 Å, against (1) half-map 1, (2) half-map 2; (3) unprocessed map and (4) final map. Blue line corresponds to FSC of model vs the corresponding asymmetric cryo-EM reconstruction and orange line to FSC of model vs the corresponding octahedrally-symmetrized cryo-EM reconstruction.
