# Supplementary material for: 2.7 Å cryo-EM structure of vitrified M. musculus H-chain apoferritin from a compact 200 keV cryo-microscope
Source: PLoS One. 2020 May 6;15(5):e0232540. doi: 10.1371/journal.pone.0232540 (PMC7202636; doi:10.1371/journal.pone.0232540)
Supplement: S5 Fig — (A) Fourier shell correlation plot for the derived 3D reconstruction. At an FSC of a reported correlation value of 0.143, resolution reaches 3.1 Å. (B) Boxplot showing the distribution of 836 calculated point spread function (PSF) resolution; the cross marks the average, whereas the black bar shows the median. (C) Percentages of PSF resolution in different ranges (a-j). (D) Histogram and directional FSC plot as calculated by 3DFSC. (E) Fourier Shell correlation of derived apoferritin model against (a) full map; (b) half-map (1) and (c) half-map (2); (d) refined against one half-map and correlated against the other. (DOCX) [file pone.0232540.s006.docx]

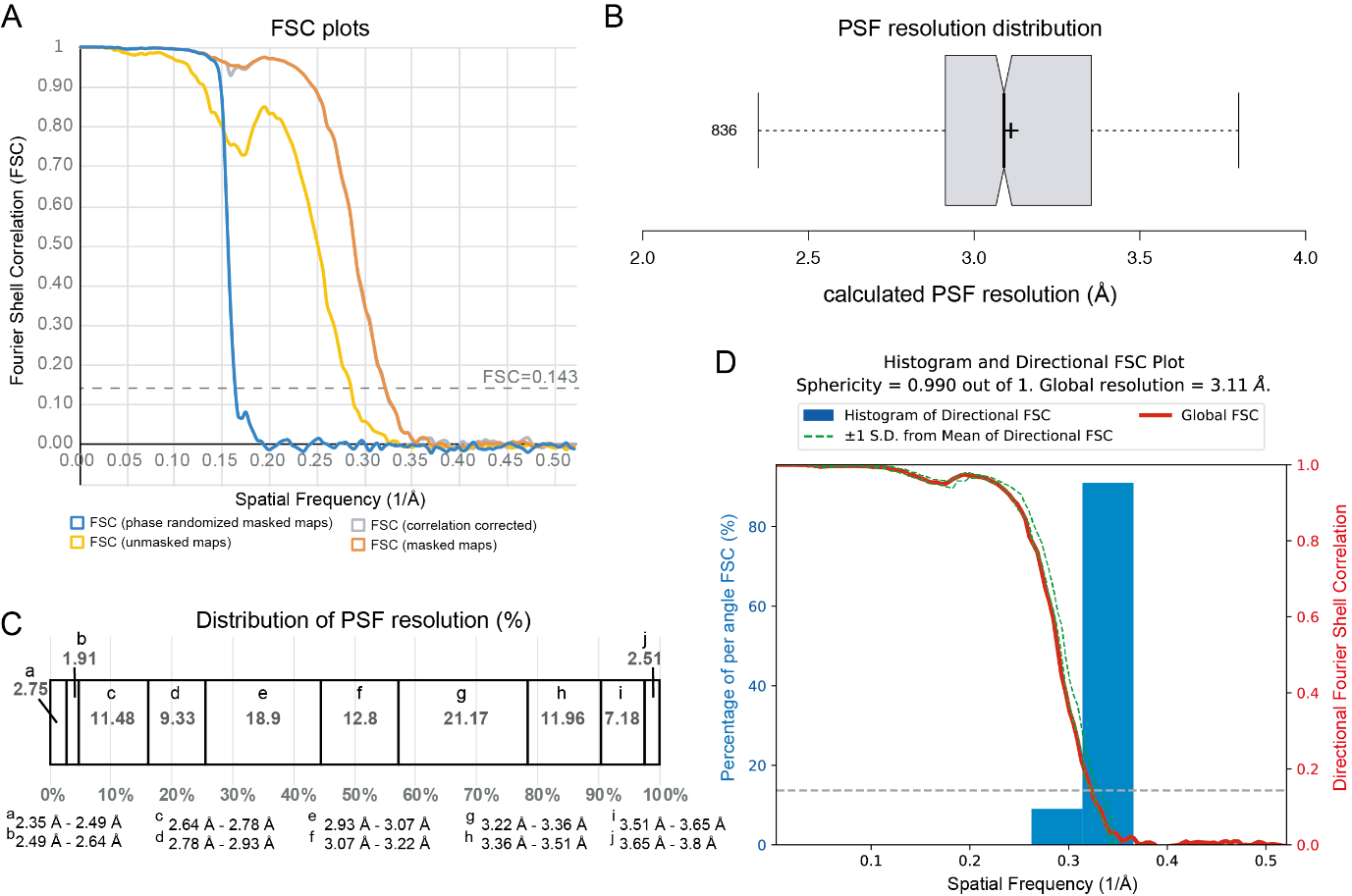
 S5 Fig. Validation metrics for apoferritin reconstructed without symmetry. (A) Fourier shell correlation plot for the derived 3D reconstruction. At an FSC of a reported correlation value of 0.143, resolution reaches 3.1 Å. (B) Boxplot showing the distribution of 836 calculated point spread function (PSF) resolution; the cross marks the average, whereas the black bar shows the median. (C) Percentages of PSF resolution in different ranges (a-j). (D) Histogram and directional FSC plot as calculated by 3DFSC. (E) Fourier Shell correlation of derived apoferritin model against (a) full map; (b) half-map (1) and (c) half-map (2); (d) refined against one half-map and correlated against the other.
