# Supplementary material for: 2.7 Å cryo-EM structure of vitrified M. musculus H-chain apoferritin from a compact 200 keV cryo-microscope
Source: PLoS One. 2020 May 6;15(5):e0232540. doi: 10.1371/journal.pone.0232540 (PMC7202636; doi:10.1371/journal.pone.0232540)
Supplement: S6 Fig — (A) Model of apoferritin in the octahedrally-reconstructed cryo-EM map, highlighting tunnel residues and densities for bound elements. (B) Recovery of bound elements of the apoferritin map, using C1 symmetry. In grey color, the symmetrized cryo-EM map is depicted; in blue, the unsymmetrized map is shown, using the same single particles. Densities for bound elements are still visible. (C) Model of apoferritin superimposed in the unsymmetrized cryo-EM map, highlighting tunnel residues and densities for bound elements. An iron cation (orange), previously found in apoferritin structures, is highlighted. (DOCX) [file pone.0232540.s007.docx]

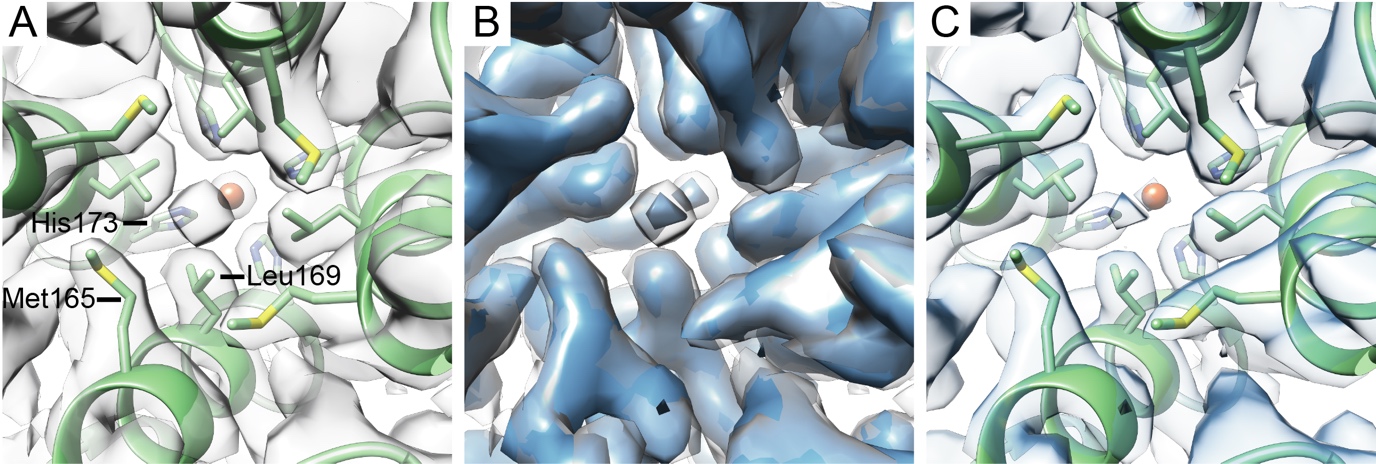


S6 Fig. Assessment of observed density in reconstruction of apoferritin using different symmetry. (A) Model of apoferritin in the octahedrally-reconstructed cryo-EM map, highlighting tunnel residues and densities for bound elements. (B) Recovery of bound elements of the apoferritin map, using C1 symmetry. In grey color, the symmetrized cryo-EM map is depicted; in blue, the unsymmetrized map is shown, using the same single particles. Densities for bound elements are still visible. (C) Model of apoferritin superimposed in the unsymmetrized cryo-EM map, highlighting tunnel residues and densities for bound elements. An iron previously found in apoferritin structures cation is highlighted (orange).
