# Supplementary figures and images for: 2.7 Å cryo-EM structure of vitrified M. musculus H-chain apoferritin from a compact 200 keV cryo-microscope
Source: PLoS One. 2020 May 6;15(5):e0232540. doi: 10.1371/journal.pone.0232540 (PMC7202636; doi:10.1371/journal.pone.0232540)

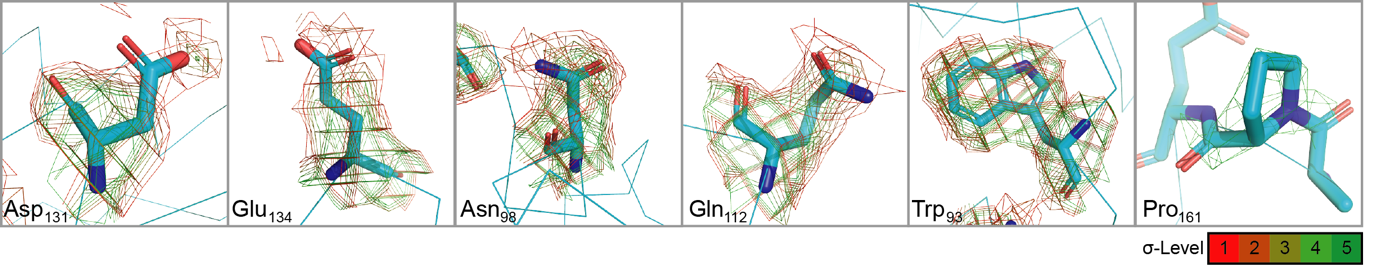
 S7 Fig. Side chains of helix E at different contour levels of the EM density (see text for details).

Supplement: S7 Fig — (DOCX) [file pone.0232540.s008.docx]
