# Supplementary material for: 2.7 Å cryo-EM structure of vitrified M. musculus H-chain apoferritin from a compact 200 keV cryo-microscope
Source: PLoS One. 2020 May 6;15(5):e0232540. doi: 10.1371/journal.pone.0232540 (PMC7202636; doi:10.1371/journal.pone.0232540)
Supplement: S8 Fig — (A) ferroxidase site (Mg2+), (B) three-fold axis channel (Mg2+) and (C) four-fold axis channel (Fe2+/Fe3+). (DOCX) [file pone.0232540.s009.docx]

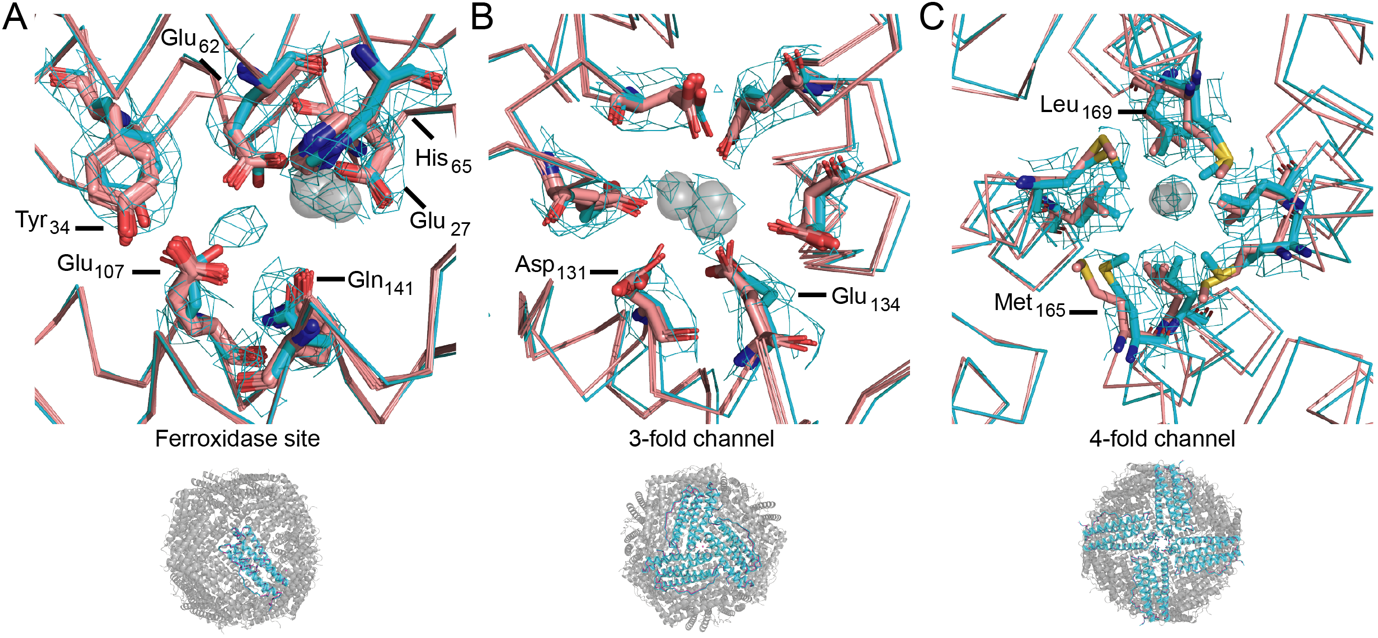


S8 Fig. Apoferritin metal ion (grey transparent spheres) binding sites in our cryo-EM reconstruction (cyan) overlaid with the crystal structure (pink). (A) ferroxidase site (Mg^2+^), (B) three-fold axis channel (Mg^2+^) and (C) four-fold axis channel (Fe^2+^/Fe^3+^).
