# Supplementary material for: 2.7 Å cryo-EM structure of vitrified M. musculus H-chain apoferritin from a compact 200 keV cryo-microscope
Source: PLoS One. 2020 May 6;15(5):e0232540. doi: 10.1371/journal.pone.0232540 (PMC7202636; doi:10.1371/journal.pone.0232540)
Supplement: S9 Fig — Briefly, a combined test specimen was used at the defocus -0.1. An image in pixel size of 0.96 was taken from the sample (S9A Fig) then the sample was slightly moved and another image at the same magnification was taken (S9B Fig). Two images were then summed pixel by pixel (S9C Fig) and the FFT of the new summed image were calculated by Thermo Fisher Scientific TIA software (S9D Fig). (A-B) Images of the combined specimen of the same region, with a slight shift of the stage; (C) Overlay of the two images (A and B) by pixel addition; (D) FFT of image (C), showing extension of the fringes to the border of the pattern. (DOCX) [file pone.0232540.s010.docx]

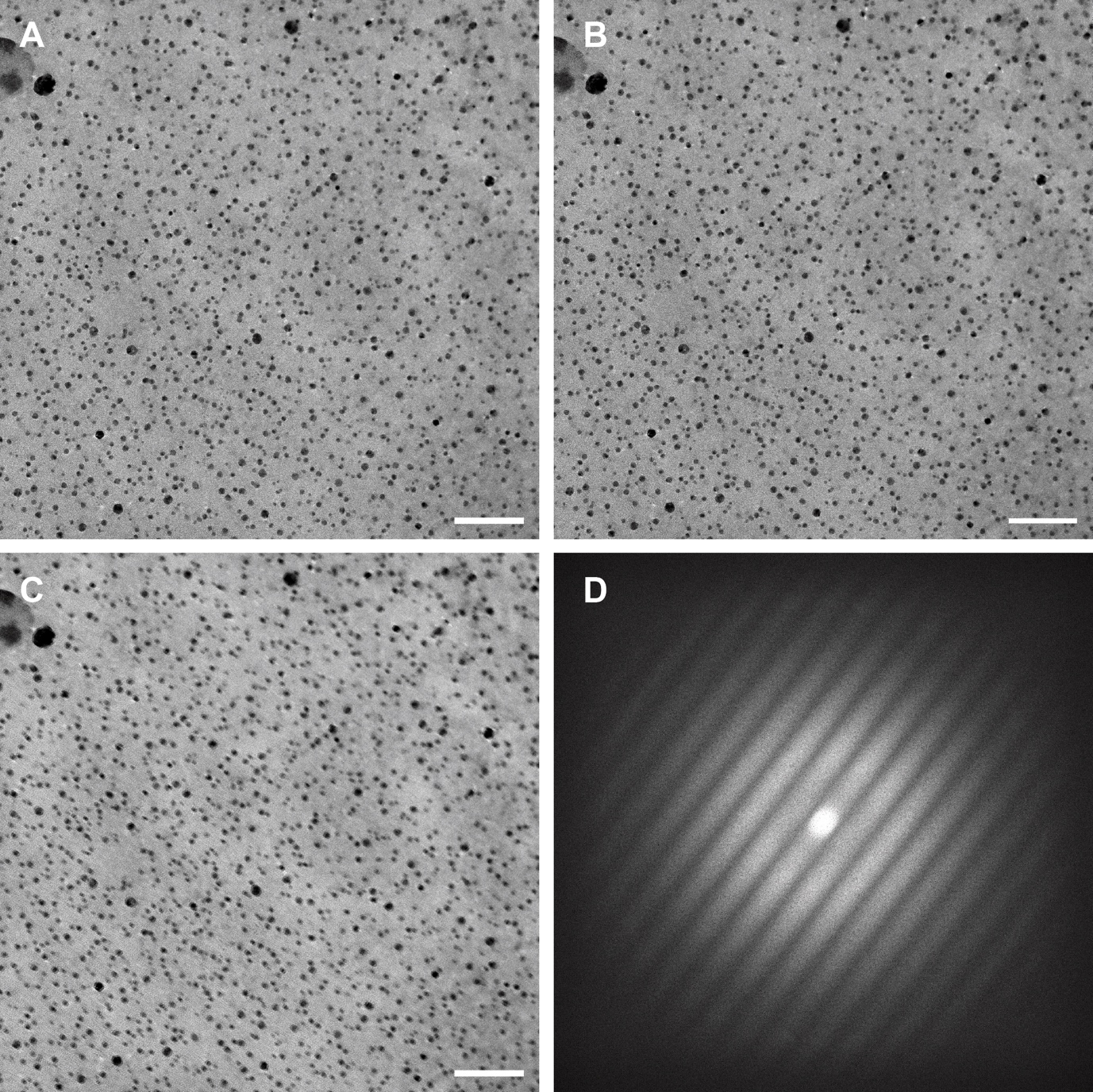
 S9 Fig. Young’s fringe on a combined test specimen to estimate the information limit of the microscope at 150kX magnification, corresponding to pixel size of 0.96 Å; Objective aperture was set to 100μm and defocus to -0.1 μm. Briefly, a combined test specimen was used at the defocus -0.1. An image in pixel size of 0.96 was taken from the sample (Fig S9A) then the sample was slightly moved and another image at the same magnification was taken (Fig S9B). Two images were then summed pixel by pixel (Fig S9C) and the FFT of the new summed image were calculated by Thermo Fisher Scientific TIA software (Fig S9D). (A-B) Images of the combined specimen of the same region, with a slight shift of the stage; (C) Overlay of the two images (A and B) by pixel addition; (D) FFT of image (C), showing extension of the fringes to the border of the pattern.
