# Supplementary material for: 2.7 Å cryo-EM structure of vitrified M. musculus H-chain apoferritin from a compact 200 keV cryo-microscope
Source: PLoS One. 2020 May 6;15(5):e0232540. doi: 10.1371/journal.pone.0232540 (PMC7202636; doi:10.1371/journal.pone.0232540)
Supplement: S1 Table — The data set described in this manuscript is highlighted in blue. (DOCX) [file pone.0232540.s011.docx]

Table S1. Cryo-EM reconstructions resolved at 200 kV < 3.0 Å. The data set described in this manuscript is highlighted in blue.

| EMDB_id | Organism/Virus | Sample | Microscope | Filter | Resolution (Å) | Detector | Particles (N) | Reference | PDB_id |
| --- | --- | --- | --- | --- | --- | --- | --- | --- | --- |
| 21024* | *Mus musculus* | apoferritin | FEI TALOS ARCTICA | None | 1.8 | GATAN K2 SUMMIT | 241878 | [4] | 6v21 |
| 9914 | *Mus musculus* | apoferritin | FEI TALOS ARCTICA | GIF | 2.0 | GATAN K2 SUMMIT | 323292 | [5] | n/a |
| 21023* | *Oryctolagus cuniculus* | aldolase | FEI TALOS ARCTICA | None | 2.1 | GATAN K2 SUMMIT | 394294 | [4] | 6v20 |
| 8743 | *Oryctolagus cuniculus* | aldolase | FEI TALOS ARCTICA | none | 2.6 | GATAN K2 SUMMIT | 83910 | [6] | 5vy5 |
| 6840 | *Escherichia coli* | beta-galactosidase with PETG | CRYOARM 200 | Omega | 2.6 | GATAN K2 IS | 93975 | n/a | n/a |
| **10205** | ***Mus musculus*** | **apoferritin** | **FEI GLACIOS^®^** | **none** | **2.7** | **FEI FALCON III** | **95733** | **n/a** | **6sht** |
| 407 | *Homo sapiens* | methemoglobin | FEI TALOS ARCTICA | none | 2.8 | GATAN K2 SUMMIT | 24308 | [7] | 6nbc, 4n7p |
| 9671 | Ao-associated virus 2 | Virus, Ao-associated virus 2 | FEI TECNAI ARCTICA | none | 2.8 | FEI FALCON II | 14434 | [8] | 6ih9 |
| 4977 | Suppressed | Suppressed | FEI TALOS ARCTICA | none | 2.8 | FEI FALCON II | 56911 | n/a | 6rpk |
| 9672 | Ao-associated virus 2 | Virus, AAV2 with AAVR | FEI TECNAI ARCTICA | none | 2.8 | FEI FALCON II | 16820 | [9] | 6ihb |
| 9608 | Seneca valley virus | Virus, Seneca valley virus | FEI TECNAI ARCTICA | none | 2.8 | FEI FALCON II | 9167 | [10] | 6adm |
| 9612 | Seneca valley virus | Virus, Seneca valley virus | FEI TECNAI ARCTICA | none | 2.8 | FEI FALCON II | 13010 | [10] | 6ads |
| 406 | *Equus caballus* | Alcohol dehydrogenase | FEI TALOS ARCTICA | none | 2.9 | GATAN K2 SUMMIT | 11672 | [7] | 6nbb, 2jhf |
| 9706 | *Macrobrachium rosenbergii* nodavirus | Virus, nodavirus | FEI TALOS ARCTICA | none | 2.9 | FEI FALCON III | 19049 | n/a | 6jjc |
| 9798 | *Sulfolobus solfataricus* | ketol-acid reductoisomerase | FEI TALOS ARCTICA | none | 2.9 | FEI FALCON III | 34743 | [10] | 6jcv |
| 20133 | *Yersinia pestis* | Lon protease | FEI TALOS ARCTICA | none | 3.0 | GATAN K2 SUMMIT | 118143 | n/a | 6on2 |
| 552 | *Homo sapiens* | m-AAA protease AFG3L2 | FEI TECNAI ARCTICA | none | 3.0 | GATAN K2 SUMMIT | 1129437 | [11] | 6nyy, 6az0 |
| 4901 | *Streptococcus sp.* | St1Cas9-sgRNA-tDNA20-AcrIIA6 | FEI TALOS ARCTICA | none | 3.0 | FEI FALCON III | 43239 | [12] | 6rja |

*These two cryo-EM maps were communicated in BioRxiv after deposition of this draft to BioRxiv and submission of the manuscript, and subsequently deposited in EMDB.
