# Supplementary material for: 2.7 Å cryo-EM structure of vitrified M. musculus H-chain apoferritin from a compact 200 keV cryo-microscope
Source: PLoS One. 2020 May 6;15(5):e0232540. doi: 10.1371/journal.pone.0232540 (PMC7202636; doi:10.1371/journal.pone.0232540)
Supplement: S2 Table — The data set described in this manuscript is highlighted in blue. (DOCX) [file pone.0232540.s012.docx]

Table S2. Reconstructions of apoferritin resolved at resolutions < 3.0 Å. The data set described in this manuscript is highlighted in blue.

| EMDB_id | Organism | Microscope | kV | Resolution (Å) | Detector | Particles (N) | References | PDB_id |
| --- | --- | --- | --- | --- | --- | --- | --- | --- |
| 9865 | *Mus musculus* | CRYOARM 300 | 300 | 1.5 | GATAN K2 SUMMIT | 120295 | n/a | n/a |
| 9599 | *Mus musculus* | FEI TITAN KRIOS | 300 | 1.6 | FEI FALCON III | 147000 | [5] |  |
| 144 | *Escherichia coli* | FEI TITAN KRIOS | 300 | 1.7 | GATAN K2 SUMMIT | 426450 | [13] | n/a |
| *21024 | *Mus musculus* | FEI TALOS ARCTICA | 200 | 1.8 | GATAN K2 SUMMIT | 241878 | [4] | n/a |
| 20026 | *Homo sapiens* | FEI TITAN KRIOS | 300 | 1.8 | GATAN K2 SUMMIT | 70648 | n/a | n/a |
| 10101 | *mus musculus* | FEI TITAN KRIOS | 300 | 1.8 | FEI FALCON III | 441902 | n/a | 6s61 |
| 9890 | *Homo sapiens* | CRYOARM 300 | 300 | 1.9 | GATAN K2 SUMMIT | 36855 | [14] | n/a |
| **9914 | *Mus musculus* | FEI TALOS ARCTICA | 200 | 2.0 | GATAN K2 SUMMIT | 323292 | [5] | n/a |
| 4905 | *Equus caballus* | FEI TITAN KRIOS | 300 | 2.1 | FEI FALCON III | 41202 | [15] | 6rjh, 4v1w |
| 4213 | *Homo sapiens* | FEI TITAN KRIOS | 300 | 2.1 | FEI FALCON III | 54006 | n/a | n/a |
| 263 | *Equus caballus* | FEI TITAN KRIOS | 300 | 2.2 | GATAN K2 SUMMIT | 65131 | [13] | n/a |
| 20027 | *Homo sapiens* | FEI TITAN KRIOS | 300 | 2.3 | GATAN K2 SUMMIT | 9600 | n/a | n/a |
| 3853 | *Homo sapiens* | FEI TITAN KRIOS | 300 | 2.5 | FEI FALCON III | 37000 | n/a | n/a |
| 20228 | *Homo sapiens* | FEI TITAN KRIOS | 300 | 2.5 | GATAN K2 SUMMIT | 304638 | [16] | 2ffx |
| 4701 | *Mus musculus* | JEOL 3200FSC | 300 | 2.7 | GATAN K2 SUMMIT | 184777 | [17] | n/a |
| **10205** | ***Mus musculus*** | **FEI GLACIOS^®^** | **200** | **2.7** | **FEI FALCON III** | **95733** | **n/a** | **6sht** |
| 4698 | *Mus musculus* | JEOL 3200FSC | 300 | 2.8 | GATAN K2 SUMMIT | 190785 | [17] | n/a |
| 20227 | *Homo sapiens* | FEI TITAN KRIOS | 300 | 2.9 | GATAN K2 SUMMIT | 96631 | [16] | 2ffx |
| 20155 | *Equus caballus* | FEI TITAN KRIOS | 300 | 2.9 | DIRECT ELECTRON DE-64 (8k x 8k) | 129140 | [18] | n/a |
| 6800 | *Homo sapiens* | FEI TITAN KRIOS | 300 | 2.9 | FEI FALCON II | 32585 | [19] | n/a |
| 20229 | *Homo sapiens* | FEI TITAN KRIOS | 300 | 2.9 | GATAN K2 SUMMIT | 73314 | [16] | 2ffx |
| 20225 | *Homo sapiens* | FEI TITAN KRIOS | 300 | 2.9 | GATAN K2 SUMMIT | 72521 | [16] | 2ffx |
| 8428 | *Equus caballus* | FEI POLARA 300 | 300 | 3.0 | GATAN K2 SUMMIT | 4300 | [20] | n/a |
| 6802 | *Homo sapiens* | FEI TITAN KRIOS | 300 | 3.0 | FEI FALCON II | 63015 | [19] | n/a |

*These two cryo-EM maps were communicated in BioRxiv after deposition of this draft to BioRxiv and submission of the manuscript, and subsequently deposited in EMDB.

******reconstruction resolved at 200 kV, an energy filter was applied with a K2 direct electron detector.
