# Supplementary material for: 2.7 Å cryo-EM structure of vitrified M. musculus H-chain apoferritin from a compact 200 keV cryo-microscope
Source: PLoS One. 2020 May 6;15(5):e0232540. doi: 10.1371/journal.pone.0232540 (PMC7202636; doi:10.1371/journal.pone.0232540)
Supplement: S3 Table — (DOCX) [file pone.0232540.s013.docx]

 Table S3. Optical presets as described in the procedure (see Supplementary Materials and Methods).

| Pre-set | Pixel Size (nm) | C1 Lens | C2  Lens* | C2 Aperture (μm) | Beam Mode** | Exposure Time (s) | Bin | Total Dose (e/Å^2^) |
| --- | --- | --- | --- | --- | --- | --- | --- | --- |
| Atlas | 91.74 | 8 | 100% | 150 | MP | 1 | 1 | Negligible |
| Grid square | 20.88 | 8 | 50% | 50 | MP | 1 | 1 | Negligible |
| Hole/EH | 0.85 | 4 | 50% | 50 | MP | 1 | 1 | 0.08 |
| Data Acquisition | 0.096 | 4 | 37.905% | 50 | NP | 30 | 1 | 30 |
| Autofocus | 0.096 | 4 | 37.905% | 50 | NP | 4 | 1 | 4 |
| Thon Rings | 0.096 | 4 | 37.905% | 50 | NP | 4 | 2 | 4 |

*The value of 37.905% is obtained as a result of parallel beam illumination adjustment

**MP: microprobe; NP: nanoprobe
